# Compliance with antibiotic therapy guidelines in french paediatric intensive care units: a multicentre observational study

**DOI:** 10.1186/s12879-024-09472-0

**Published:** 2024-06-12

**Authors:** Romain Amadieu, Camille Brehin, Adéla Chahine, Erick Grouteau, Damien Dubois, Caroline Munzer, Clara Flumian, Olivier Brissaud, Barbara Ros, Gael Jean, Camille Brotelande, Brendan Travert, Nadia Savy, Benoit Boeuf, Ghida Ghostine, Isabelle Popov, Pauline Duport, Richard Wolff, Laure Maurice, Stephane Dauger, Sophie Breinig

**Affiliations:** 1 Neonatal and Paediatric Intensive Care Unit, Children’s Hospital, Toulouse University Hospital, 330 Avenue de Grande Bretagne, TSA 70034, Toulouse Cedex 9, 31059 France; 2 Paediatric Infectious Diseases Department, Children’s Hospital, Toulouse University Hospital, Toulouse, France; 3 General Paediatrics Department, Children’s Hospital, Toulouse University Hospital, Toulouse, France; 4 Bacteriology-Hygiene Department, Toulouse University Hospital, Toulouse, France; 5 Paediatric Clinical Research Department, Children’s Hospital, Equipe MéDatAS-CIC 1436, Toulouse University Hospital, Toulouse, France; 6 Neonatal and Paediatric Intensive Care Unit, Pellegrin University Hospital, Bordeaux University, Bordeaux, France; 7 Paediatric Intensive Care Unit, Arnaud de Villeneuve University Hospital, Montpellier University, Montpellier, France; 8 Neonatal and Paediatric Intensive Care Unit, Mère-Enfant University Hospital, Nantes University, Nantes, France; 9 Neonatal and Paediatric Intensive Care Unit, Estaing University Hospital, Clermont-Ferrand University, Clermont-Ferrand, France; 10 Neonatal and Paediatric Intensive Care Unit, Amiens-Picardie University Hospital, Amiens University, Amiens, France; 11 Neonatal and Paediatric Intensive Care Unit, Felix Guyon University Hospital, La Réunion University, Saint-Denis, Ile de la Réunion, France; 12 Paediatric Intensive Care Unit, Robert Debré University Hospital, Assistance Publique-Hôpitaux de Paris, Paris University, Paris, France

**Keywords:** Children, Paediatric intensive care unit, Antibiotic therapy, Compliance, Guidelines, Antimicrobial stewardship programme

## Abstract

**Background:**

Bacterial infections (BIs) are widespread in ICUs. The aims of this study were to assess compliance with antibiotic recommendations and factors associated with non-compliance.

**Methods:**

We conducted an observational study in eight French Paediatric and Neonatal ICUs with an antimicrobial stewardship programme (ASP) organised once a week for the most part. All children receiving antibiotics for a suspected or proven BI were evaluated. Newborns < 72 h old, neonates < 37 weeks, age ≥ 18 years and children under surgical antimicrobial prophylaxis were excluded.

**Results:**

139 suspected (or proven) BI episodes in 134 children were prospectively included during six separate time-periods over one year. The final diagnosis was 26.6% with no BI, 40.3% presumed (i.e., not documented) BI and 35.3% documented BI. Non-compliance with antibiotic recommendations occurred in 51.1%. The main reasons for non-compliance were inappropriate choice of antimicrobials (27.3%), duration of one or more antimicrobials (26.3%) and length of antibiotic therapy (18.0%). In multivariate analyses, the main independent risk factors for non-compliance were prescribing ≥ 2 antibiotics (OR 4.06, 95%CI 1.69–9.74, *p* = 0.0017), duration of broad-spectrum antibiotic therapy ≥ 4 days (OR 2.59, 95%CI 1.16–5.78, *p* = 0.0199), neurologic compromise at ICU admission (OR 3.41, 95%CI 1.04–11.20, *p* = 0.0431), suspected catheter-related bacteraemia (ORs 3.70 and 5.42, 95%CIs 1.32 to 15.07, *p* < 0.02), a BI site classified as “other” (ORs 3.29 and 15.88, 95%CIs 1.16 to 104.76, *p* < 0.03), sepsis with ≥ 2 organ dysfunctions (OR 4.21, 95%CI 1.42–12.55, *p* = 0.0098), late-onset ventilator-associated pneumonia (OR 6.30, 95%CI 1.15–34.44, *p* = 0.0338) and ≥ 1 risk factor for extended-spectrum β-lactamase-producing Enterobacteriaceae (OR 2.56, 95%CI 1.07–6.14, *p* = 0.0353). Main independent factors for compliance were using antibiotic therapy protocols (OR 0.42, 95%CI 0.19–0.92, *p* = 0.0313), respiratory failure at ICU admission (OR 0.36, 95%CI 0.14–0.90, *p* = 0.0281) and aspiration pneumonia (OR 0.37, 95%CI 0.14–0.99, *p* = 0.0486).

**Conclusions:**

Half of antibiotic prescriptions remain non-compliant with guidelines. Intensivists should reassess on a day-to-day basis the benefit of using several antimicrobials or any broad-spectrum antibiotics and stop antibiotics that are no longer indicated. Developing consensus about treating specific illnesses and using department protocols seem necessary to reduce non-compliance. A daily ASP could also improve compliance in these situations.

**Trial Registration:**

ClinicalTrials.gov: number NCT04642560. The date of first trial registration was 24/11/2020.

**Supplementary Information:**

The online version contains supplementary material available at 10.1186/s12879-024-09472-0.

## Background

Bacterial infections (BIs) are widespread in Paediatric and Neonatal Intensive Care Units (ICUs) and 30–61% of neonates and children hospitalised in ICUs receive antibiotic treatments [1, 2]. For suspected sepsis or septic shock, the Surviving Sepsis Campaign (SSC) recommends the early administration of empiric broad-spectrum antibiotic therapy with one or more antimicrobials to cover all likely pathogens, appropriate routine microbiologic cultures (including blood) before starting antibiotic therapy if doing so causes no substantial delay in starting antimicrobials as well as early de-escalation of antimicrobials based on culture results, susceptibility results and clinical improvement [3, 4]. The choice of antimicrobials, number of antibiotic doses in 24 h, daily dose and duration are determined by local epidemiology, patient characteristics (age, patient history, allergies, multidrug-resistant [MDR] status), BI characteristics (infection site(s), community or hospital-acquired infection, microbiological results) as well as clinical and biological evolution in line with published guidelines. Their recommendations are numerous: French (SPILF, GPIP, SFAR, SRLF, HAS) [5–21], European (ESCMID) [22, 23] and American (IDSA) [24–28] guidelines. Long courses of antibiotic therapy and broad-spectrum antimicrobials increase the length of hospitalisation and are associated with changes in the microbiome [29], emergence of multidrug resistant organisms [30, 31] and antibiotic-associated adverse events (toxicity, overdose, allergy) [32]. On the other hand, too short antibiotic therapy can expose the patient to a risk of BI recurrence. In 2017 and 2021, the Société de Pathologie Infectieuse de Langue Française (SPILF or French Language Society for Infectious Diseases) and Groupe de Pathologies Infectieuses en Pédiatrie (GPIP or French Group for Paediatric Infectious Diseases) published French recommendations for the shortest treatment durations for BIs [5, 6]. To our knowledge, compliance with these recommendations has never been evaluated.

In this context, we assessed compliance with recommendations for antibiotic prescriptions, and factors associated with non-compliance for children hospitalised in ICU and receiving systemic antibiotics for an episode of suspected or proven BI.

## Methods

### Study design, setting and participants

We conducted an observational, prospective, multicentre study in eight French Paediatric and Neonatal ICUs (Appendix 1) during six separate time-periods between June 2020 and May 2021. After an inclusion period testing the feasibility of data collection at the Coordinator Centre (Toulouse) from June to August 2020, we then arbitrarily chose a priori five weeks each spread two months apart for multicentre inclusion over the course of one year. All participating ICUs had the possibility of an audit with an infectious disease specialist over the telephone and most had an antimicrobial stewardship programme (ASP) organised in once weekly multidisciplinary (intensivists, microbiologists and paediatric infectious disease specialists) staff meetings with prospective audit and feedback. Intensivists were the only prescribers. This study was supported by the Groupe Francophone de Réanimation et d’Urgences Pédiatriques (GFRUP or French-Speaking Group for Paediatric Intensive and Emergency Care). All methods were performed in accordance with relevant guidelines and regulations. In accordance with French Ethics and Regulatory Law (Public Health Code), this trial is covered by reference methodology MR-004 from the French Data Protection Commission (CNIL). It was approved by Toulouse University Hospital and is registered on its Study Data Register under number RnIPH2019-79 and on the ClinicalTrials.gov website under number NCT04642560. The date of first trial registration on ClinicalTrials.gov was 24/11/2020.

During the study periods, all consecutive neonates and children hospitalised in ICUs and receiving systemic (intravascular, intramuscular or oral) antibiotic treatment for a suspected or proven community-acquired or nosocomial BI were assessed for eligibility. Antibiotics had to be initiated in ICUs during the study periods or no more than 24 h prior to ICU admission occurring over the study periods. We called this episode “first suspected or proven BI episode” to distinguish it from a possible BI recurrence at a later stage. Exclusion criteria were: newborns < 72 h old; neonates < 37 weeks post-menstrual age; age ≥ 18 years; children under surgical antimicrobial prophylaxis; and children previously included in an ongoing interventional study. Informed verbal consent was obtained from the parents or legal guardians of the patient prior to study enrolment.

### Data collection

Data on patient characteristics and the first suspected or proven BI episode (characteristics, organ dysfunction scores, antibiotic therapy and concomitant therapeutics other than antibiotics) were prospectively collected on a daily basis by medical study site investigators so long as the patient was receiving antibiotics in hospital for the first suspected or proven BI episode (ICU and general paediatric ward if antibiotic therapy was not completed in the ICU) (Appendix 2). D0 was the day of antibiotic therapy initiation. Additional data on the length of ICU and hospital stay, mortality, outpatient antibiotic therapy (if this was the case) and recurrence of BI occurring within 28 days following D0 were registered upon hospital discharge. Antibiotics used for any other infections during the 28 days following D0 and surgical antimicrobial prophylaxis were not taken into account.

The primary endpoint was the number of first episodes for which antibiotics were prescribed inappropriately on the basis of current recommendations (non-compliance) involving one or more of the following parameters: length of antibiotic therapy, duration of each antimicrobial treatment, choice of antimicrobials, number of antibiotic doses in 24 h, daily dose of antibiotic therapy and reassessment of antibiotic therapy at 72 h. Secondary endpoints were: number of first episodes with non-compliance for any of the parameters; length of antibiotic therapy for the first suspected or proven BI episode; duration of broad-spectrum antibiotic therapy for the first suspected or proven BI episode; and recurrence of BI within 28 days following D0.

Recurrence of infection was defined as the isolation of one or more of the initial causative bacteria from the same or another site at 48 h or more after cessation of antibiotics, combined with clinical signs or symptoms of infection or the need to prescribe a new antimicrobial therapy covering this pathogen [33]. Only recurrences during the same hospital stay as for the first BI episode and occurring within 28 days following D0 were taken into consideration.

Finally, we used two separate definitions to identify broad-spectrum antibiotics: the standard definition [34] and the 2019 AWaRe (Access, Watch, Reserve) classification (Watch and Reserve groups) [35, 36].

### Analysis of compliance with antibiotic recommendations

For each first suspected or proven BI episode, the same paediatric infectious disease expert committee (CB and EG) analysed compliance with antibiotic recommendations for length of antibiotic therapy, duration of each antimicrobial treatment, choice of antimicrobials, number of antibiotic doses in 24 h, daily dose of antibiotic therapy and reassessment of antibiotic therapy at 72 h (Appendix 2 and 3). The two experts relied principally on the SPILF and GPIP French guidelines [5, 6] for the duration of antibiotic therapy and each antimicrobial treatment and on a combination of French, European and American guidelines [5–28] for the choice of antimicrobials, number of antibiotic doses in 24 h and daily dose of antibiotic therapy. Non-compliance (inappropriate antibiotic prescription based on the guidelines) was only determined if both experts agreed. To detect a high background rate of resistant pathogens in ICUs which would justify the use of initial broad-spectrum empiric therapy during the study period, we asked participating centres for their local microbiological data from the two years prior to the study period (2018 and 2019).

### Statistical analysis

The results of descriptive statistics were presented as absolute frequencies (%) for qualitative variables and as medians (IQR) for continuous variables.

For all first suspected (or proven) BI episodes and also for only first confirmed (documented or not) BI episodes, we performed univariate analyses to assess factors that might be associated with non-compliance for each parameter and for all parameters combined. For independent qualitative variables, we used the χ² test or Fisher’s exact test while independent quantitative variables employed a two-sample t-test or a Wilcoxon-Mann-Whitney rank-based test. To convert quantitative variables into classes when necessary, we determined the thresholds using Youden’s index.

To establish the independent predictive factors for non-compliance, we then carried out multivariate analyses by stepwise logistic regression after selecting the independent qualitative variables associated with the dependent variable with *p* < 0.20. The association between variables was significant if *p* < 0.05. Odds ratios (ORs) and 95% confidence intervals (CIs) were calculated for the significant variables. When one or more centres demonstrated significance during the multivariate analysis, ORs were adjusted for each centre.

Additionally, to evaluate if the use of an ASP during a suspected (or proven) BI episode is a factor modifying the effect of independent variables on non-compliance, we conducted ASP-stratified univariate logistic regression analyses of all factors potentially associated with non-compliance with recommendations for all parameters and for each parameter (length of antibiotic therapy, duration of each antimicrobial treatment, choice of antimicrobials, daily dose of antibiotic therapy and reassessment of antibiotic therapy at 72 h). The ASP sub-group included all episodes that received auditing with an antimicrobial stewardship team for antibiotic duration and/or choice of antimicrobials. Stratified ORs and 95% CIs were calculated for all independent variables in both the ASP and non-ASP sub-groups and were compared with ORs and 95% CIs of all suspected (or proven) BI episodes.

All statistical analyses were performed using SAS software (version 9.4, Cary, NC, USA).

## Results

### Local microbiological data of participating intensive care units

Local ICU microbiological data from the two years prior to the study period (years 2018–2019) were available at 5 of the 8 centres (Appendix 4). Isolation frequencies for major pathogens was quite similar between the centres. Resistance rates to major antibiotics (intermediate or resistance categories) remained at a low background rate overall except for Enterobacterales resistance to third-generation cephalosporins in some centres.

### Population characteristics

During the study periods, 868 children were hospitalised in the eight participating ICUs. One hundred thirty-nine first suspected (or proven) BI episodes occurring in 134 children were included. All children meeting the entire inclusion criteria and with no non-inclusion criteria were included. No one was missed and none of the children had legal guardians who refused consent. The median age was 0.8 (IQR 0.1–6.3) years and 20.1% of episodes affected neonates. Patient characteristics, infection characteristics and antibiotic therapy for first suspected (or proven) BI episodes, and outcomes are set out in Table 1. The most frequent initially suspected BI sites were respiratory (56.1%), catheter-related bacteraemia (20.1%) and intra-abdominal (9.4%). Final diagnosis was no BI for 26.6% of the episodes, presumed (i.e., not documented) BI for 40.3% and documented BI for 35.3%. Three episodes combined both presumed and documented BI. The median length of antibiotic therapy for the first suspected (or proven) BI episode was 7.1 (IQR 4.0-10.5) days. Recurrence occurred for only two episodes (1.4%).

**Table 1:** Patient characteristics, infection characteristics and antibiotic therapy for first suspected (or proven) bacterial infection episodes, and outcome: univariate analysis of factors associated with non-compliance with antibiotic recommendations for all parameters

Characteristics	All Episodes (*n* = 139)	Episodes in children with compliance with recommendations for all parameters (*n* = 65)^a^	Episodes in children with non-compliance with recommendations for all parameters (*n* = 68)^a^	*p* value
Number of children	134	64	68	—
**Patient characteristics**				
Age at Day 0 (years), median (IQR)	0.8 (0.1–6.3)	0.6 (0.1–5.3)	0.8 (0.1–5.8)	0.5978
Neonates, n (%)	28 (20.1)	14 (21.5)	14 (20.6)	0.8931
Weight at Day 0 (kg), median (IQR)	8.0 (3.7–20.0)	7.4 (3.4–18.0)	8.4 (3.8–19.0)	0.3497
Male sex, n (%)	70 (50.4)	29 (44.6)	36 (52.9)	0.3370
Reason for admission to ICU, n (%)				
Trauma	14 (10.1)	7 (10.8)	5 (7.4)	0.4918
Postoperative recovery	15 (10.8)	6 (9.2)	9 (13.2)	0.4655
Burns	1 (0.7)	0 (0.0)	1 (1.5)	1.0000
Infection	22 (15.8)	6 (9.2)	14 (20.6)	**0.0670**
Respiratory failure	35 (25.2)	25 (38.5)	10 (14.7)	**0.0019**
Cardiovascular compromise	24 (17.3)	9 (13.8)	14 (20.6)	0.3041
Neurologic compromise	17 (12.2)	6 (9.2)	11 (16.2)	0.2304
Acute kidney injury	2 (1.4)	2 (3.1)	0 (0.0)	0.2370
Gastrointestinal disease	5 (3.6)	2 (3.1)	2 (2.9)	1.0000
Liver disease	1 (0.7)	0 (0.0)	1 (1.5)	1.0000
Metabolic derangement/drug poisoning	1 (0.7)	0 (0.0)	1 (1.5)	1.0000
Haematologic derangement	1 (0.7)	1 (1.5)	0 (0.0)	0.4887
Other	1 (0.7)	1 (1.5)	0 (0.0)	0.4887
PIM-2 score at ICU admission, median (IQR)	3.2 (1.1–8.7)	2.8 (1.1–7.3)	3.7 (1.1–8.3)	0.8275
Category at Day 0, n (%)				
Medical	87 (62.6)	43 (66.2)	40 (58.8)	—
Surgical	52 (37.4)	22 (33.8)	28 (41.2)	0.3830
MDR status, n (%)				
MDR bacteria colonisation or previous infection	7 (5.0)	2 (3.1)	5 (7.4)	0.4413
ESBL Enterobacteriaceae colonisation or previous infection	4 (2.9)	1 (1.5)	3 (4.4)	0.6196
MRSA colonisation or previous infection	3 (2.2)	1 (1.5)	2 (2.9)	1.0000
≥1 risk factor for ESBL Enterobacteriaceae^b^	41 (29.5)	16 (24.6)	22 (32.4)	0.3235
≥1 risk factor for MRSA^c^	5 (3.6)	2 (3.1)	3 (4.4)	1.0000
Patient context for onset of the first suspected (or proven) bacterial infection episode, n (%)				
Trauma	14 (10.1)	7 (10.8)	5 (7.4)	0.4918
Postoperative recovery	43 (30.9)	18 (27.7)	24 (35.3)	0.3458
Burns	2 (1.4)	1 (1.5)	1 (1.5)	1.0000
Respiratory failure	28 (20.1)	21 (32.3)	7 (10.3)	**0.0019**
Cardiovascular compromise	16 (11.5)	7 (10.8)	8 (11.8)	0.8560
ECMO	3 (2.2)	1 (1.5)	2 (2.9)	1.0000
Neurologic compromise	15 (10.8)	6 (9.2)	9 (13.2)	0.4655
Renal replacement therapy	2 (1.4)	2 (3.1)	0 (0.0)	0.2370
Hepatic failure	1 (0.7)	0 (0.0)	1 (1.5)	1.0000
Metabolic derangement/drug poisoning	1 (0.7)	0 (0.0)	1 (1.5)	1.0000
Hematologic derangement	3 (2.2)	1 (1.5)	1 (1.5)	1.0000
Malaria	1 (0.7)	0 (0.0)	1 (1.5)	1.0000
None	16 (11.5)	4 (6.2)	11 (16.2)	**0.0678**
**Characteristics of the first suspected (or proven) bacterial infection episode**				
Initially suspected (or proven) bacterial infection site(s), n (%)				
Primary bacteraemia	8 (5.8)	4 (6.2)	4 (5.9)	1.0000
Catheter-related bacteraemia	28 (20.1)	8 (12.3)	19 (27.9)	**0.0251**
Ear-Nose-Throat	3 (2.2)	0 (0.0)	3 (4.4)	0.2447
Respiratory	78 (56.1)	40 (61.5)	34 (50.0)	**0.1806**
Community-acquired pneumonia	21 (15.1)^d^	13 (20.0)^e^	8 (11.8)	**0.1929**
Atypical community-acquired pneumonia	4 (2.9)^d^	4 (6.2)^e^	0 (0.0)	**0.0543**
Aspiration pneumonia	30 (21.6)^d^	16 (24.6)^e^	10 (14.7)	**0.1497**
Pneumonia with parapneumonic pleural effusion	2 (1.4)^d^	1 (1.5)^e^	1 (1.5)	1.0000
Pleural empyema	1 (0.7)^d^	1 (1.5)^e^	0 (0.0)	0.4887
Non-ventilator HAP	8 (5.8)^d^	2 (3.1)^e^	6 (8.8)	0.2749
Early-onset VAP	5 (3.6)^d^	2 (3.1)^e^	3 (4.4)	1.0000
Late-onset VAP	9 (6.5)^d^	2 (3.1)^e^	6 (8.8)	0.2749
Intra-abdominal	13 (9.4)	4 (6.2)	7 (10.3)	0.3862
Urinary tract	7 (5.0)	3 (4.6)	4 (5.9)	1.0000
Central nervous system	12 (8.6)	4 (6.2)	8 (11.8)	0.2589
Skin and soft tissue	7 (5.0)	2 (3.1)	5 (7.4)	0.4413
Bones and joints	1 (0.7)	1 (1.5)	0 (0.0)	0.4887
Cardiovascular	3 (2.2)	2 (3.1)	1 (1.5)	0.6137
Other	25 (18.0)	10 (15.4)	15 (22.1)	0.3247
PIMS	3 (2.2)^f^	2 (3.1)	1 (1.5)^g^	0.6137
Deep surgical site infection, not elsewhere classified	2 (1.4)^f^	2 (3.1)	0 (0.0)^g^	0.2370
Open fracture	4 (2.9)^f^	0 (0.0)	4 (5.9)^g^	**0.1197**
Late-onset neonatal bacterial infection	11 (7.9)^f^	5 (7.7)	6 (8.8)^g^	0.8128
Final diagnosis, n (%)				
No bacterial infection	37 (26.6)	24 (36.9)	13 (19.1)	**0.0220**
Presumed bacterial infection	56 (40.3)^h^	23 (35.4)	31 (45.6)^i^	0.2310
Documented bacterial infection	49 (35.3)^h^	18 (27.7)	26 (38.2)^i^	**0.1964**
Organ dysfunction scores	(*n* = 136)	(*n* = 64)	(*n* = 66)	
PELOD-2 at Day 0, median (IQR)	3.0 (0.5-6.0)	3.0 (0.5-5.0)	3.0 (0.0–6.0)	0.5969
PELOD-2 at Day 0 ≥ 6, n (%)	35 (25.7)	12 (18.8)	19 (28.8)	**0.1794**
pSOFA at Day 0, median (IQR)	3.0 (1.0–5.0)	2.0 (1.0–5.0)	3.0 (1.0–5.0)	0.6863
Maximum PELOD-2, median (IQR)	3.0 (1.0–6.0)	3.0 (1.0–5.0)	3.0 (1.0–6.0)	0.2445
Maximum PELOD-2 ≥ 6, n (%)	40 (29.4)	13 (20.3)	22 (33.3)	**0.0943**
Maximum pSOFA, median (IQR)	3.0 (1.0–6.0)	3.0 (1.0–5.0)	4.0 (1.0–6.0)	0.2619
Maximum pSOFA ≥ 4, n (%)	66 (48.5)	25 (39.1)	35 (53.0)	**0.1102**
**Antibiotic therapy for the first suspected (or proven) bacterial infection episode**				
Number of antimicrobials used per episode ≥ 2, n (%)	97 (69.8)	35 (53.8)	57 (83.8)	**0.0002**
Length of antibiotic therapy (days), median (IQR)	7.1 (4.0-10.5)	6.2 (2.5–9.9)	7.9 (5.4–11.1)	**0.0246**
Length of antibiotic therapy ≥ 8 days, n (%)	55 (39.6)	23 (35.4)	32 (47.1)	**0.1786**
Duration of broad-spectrum antibiotic therapy (days), based on the standard definition, median (IQR)	4.9 (2.0-8.2)	3.0 (1.5–7.7)	6.5 (3.0–10.0)	**0.0117**
Duration of broad-spectrum antibiotic therapy, based on the standard definition ≥ 4 days, n (%)	80 (57.6)	30 (46.2)	48 (70.6)	**0.0042**
Duration of broad-spectrum antibiotic therapy (days), according to the AWaRe classification (Watch and Reserve antibiotics), median (IQR)	3.3 (0.0-7.9)	1.8 (0.0-7.7)	4.5 (0.7–8.1)	**0.0812**
Duration of broad-spectrum antibiotic therapy, according to the AWaRe classification (Watch and Reserve antibiotics) ≥ 3 days, n (%)	73 (52.5)	25 (38.5)	46 (67.6)	**0.0007**
Audit with an antimicrobial stewardship team, n (%)				
for antibiotic duration	60 (43.2)	28 (43.1)	30 (44.1)	0.9037
for choice of antimicrobials	58 (41.7)	26 (40.0)	30 (44.1)	0.6307
Department protocol, n (%)				
for antibiotic duration	55 (39.6)	35 (53.8)	19 (27.9)	**0.0024**
for choice of antimicrobials	58 (41.7)	36 (55.4)	21 (30.9)	**0.0043**
**Outcome**				
Recurrence of bacterial infection, n (%)	2 (1.4)	0 (0.0)	2 (2.9)	0.4965
Length of ICU stay from Day 0 (days), median (IQR)	5 (2–8)	5 (3–8)	5 (2–9)	0.7572
Length of hospital stay from Day 0 (days), median (IQR)	10 (6–22)	10 (6–22)	11.5 (6–25)	0.5205
Death, n (%)	15 (10.8)	5 (7.7)	4 (5.9)	0.7406
Death related to sepsis, n (%)	3 (2.2)	0 (0.0)	0 (0.0)	—

Data are number of episodes (%) or median (IQR). P values were determined by χ² tests or Fisher’s exact tests for independent qualitative variables and two-sample t-tests or Wilcoxon-Mann-Whitney rank-based tests for independent quantitative variables. To convert quantitative variables into classes when necessary, we determined the thresholds using the Youden’s index. Day 0 is the day of antibiotic therapy initiation

AWaRe: Access, Watch, Reserve; ECMO: Extracorporeal Membrane Oxygenation; ESBL: Extended-Spectrum β-Lactamase producing; HAP: Hospital-Acquired Pneumonia; ICU: Intensive Care Unit; MDR: Multidrug-Resistant; MRSA: Methicillin-Resistant *Staphylococcus aureus*; PELOD-2: Pediatric Logistic Organ Dysfunction-2; PIM-2: Paediatric Index of Mortality-2; PIMS: Paediatric Inflammatory Multisystem Syndrome temporally associated with SARS-CoV-2 infection; pSOFA: Pediatric Sequential Organ Failure Assessment; VAP: Ventilator-Associated Pneumonia

^a^Six suspected or proven bacterial infection episodes were not assessable because of death. ^b^Risk factors for ESBL Enterobacteriaceae: antibiotic therapy with Amoxicillin-clavulanic acid, 2nd or 3rd generation cephalosporin, fluoroquinolone (including single dose) or Piperacillin-tazobactam in the past 3 months, travel in endemic ESBL Enterobacteriaceae areas within the previous 3 months, living in a long-term facility and having an indwelling catheter and/or gastrostomy. ^c^Risk factors for MRSA: MRSA colonisation or previous infection, chronic skin lesions, chronic renal replacement therapy. ^d^80 respiratory infection sites were initially suspected or proven in 78 episodes. ^e^41 respiratory infection sites were initially suspected or proven in 40 episodes. ^f^28 other infection sites were initially suspected or proven in 25 episodes. ^g^18 other infection sites were initially suspected or proven in 15 episodes. ^h^Three episodes combined both presumed and documented bacterial infection. ^i^Two episodes combined both presumed and documented bacterial infection

Diagnosis of BI (documented or not) was confirmed for 102 of the 139 first suspected BI episodes (Table 2). The 2005 IPSC infection severity was as follows: 25.5% infection without sepsis; 52.0% sepsis (excluding severe sepsis and septic shock); 11.8% severe sepsis (excluding septic shock); and 10.8% septic shock. Among the 76 episodes of sepsis, severe sepsis or septic shock, respiratory dysfunction was the most common acute organ dysfunction related to the BI and found in 60.5% of the episodes. The most frequent BI sites ultimately identified were respiratory (57.8%), mainly community-acquired pneumonia (13.7%) and aspiration pneumonia (23.5%), catheter-related bacteraemia (18.6%) and intra-abdominal (10.8%).

**Table 2:** Characteristics of first episodes of confirmed (documented or not) bacterial infection: univariate analysis of factors associated with non-compliance with antibiotic recommendations for all parameters

Characteristics	All Bacterial infection episodes (*n* = 102)	Bacterial infection episodes in children with compliance with recommendations for all parameters (*n* = 41)^a^	Bacterial infection episodes in children with non-compliance with recommendations for all parameters (*n* = 55)^a^	*p* value
Origin of the first bacterial infection episode, n (%)				
Community	56 (54.9)^b^	26 (63.4)^c^	27 (49.1)^d^	**0.1627**
Nosocomial	50 (49.0)^b^	16 (39.0)^c^	30 (54.5)^d^	**0.1321**
Severity of the first bacterial infection episode^e^, n (%)				0.4283
Infection without sepsis	26 (25.5)	9 (22.0)	17 (30.9)	—
Sepsis	53 (52.0)	25 (61.0)	26 (47.3)	—
Severe sepsis	12 (11.8)	3 (7.3)	8 (14.5)	—
Septic shock	11 (10.8)	4 (9.8)	4 (7.3)	—
Acute organ dysfunctions related to the first bacterial infection episode^e^ (only for sepsis, severe sepsis and septic shock), n (%)	(*n* = 76)	(*n* = 32)	(*n* = 38)	
Cardiovascular	11 (14.5)	4 (12.5)	4 (10.5)	1.0000
Respiratory	46 (60.5)	20 (62.5)	22 (57.9)	0.6952
ARDS	5 (6.6)	2 (6.3)	2 (5.3)	1.0000
Neurologic	4 (5.3)	2 (6.3)	2 (5.3)	1.0000
Hematologic	8 (10.5)	2 (6.3)	4 (10.5)	0.6809
Renal	12 (15.8)	2 (6.3)	8 (21.1)	**0.0970**
Hepatic	7 (9.2)	1 (3.1)	4 (10.5)	0.3662
Number of acute organ dysfunctions related to the first bacterial infection episode^e^ (only for sepsis, severe sepsis and septic shock), n (%)	(*n* = 76)	(*n* = 32)	(*n* = 38)	
≥1	54 (71.1)	24 (75.0)	26 (68.4)	0.5439
≥2	17 (22.4)	3 (9.4)	10 (26.3)	**0.0694**
≥3	9 (11.8)	2 (6.3)	5 (13.2)	0.4415
≥4	6 (7.9)	2 (6.3)	2 (5.3)	1.0000
≥5	2 (2.6)	0 (0.0)	1 (2.6)	1.0000
Bacterial infection site(s) ultimately identified for the first bacterial infection episode, n (%)				
Primary bacteraemia	4 (3.9)	1 (2.4)	3 (5.5)	0.6334
Catheter-related bacteraemia	19 (18.6)	6 (14.6)	12 (21.8)	0.3724
Ear-Nose-Throat	2 (2.0)	0 (0.0)	2 (3.6)	0.5055
Respiratory	59 (57.8)	27 (65.9)	29 (52.7)	**0.1969**
Community-acquired pneumonia	14 (13.7)^f^	8 (19.5)	6 (10.9)	0.2374
Atypical community-acquired pneumonia	1 (1.0)^f^	1 (2.4)	0 (0.0)	0.4271
Aspiration pneumonia	24 (23.5)^f^	13 (31.7)	8 (14.5)	**0.0442**
Pneumonia with parapneumonic pleural effusion	1 (1.0)^f^	0 (0.0)	1 (1.8)	1.0000
Pleural empyema	1 (1.0)^f^	1 (2.4)	0 (0.0)	0.4271
Non-ventilator HAP	8 (7.8)^f^	2 (4.9)	6 (10.9)	0.4599
Early-onset VAP	3 (2.9)^f^	1 (2.4)	2 (3.6)	1.0000
Late-onset VAP	8 (7.8)^f^	1 (2.4)	6 (10.9)	0.2327
Intra-abdominal	11 (10.8)	4 (9.8)	5 (9.1)	1.0000
Urinary tract	7 (6.9)	3 (7.3)	4 (7.3)	1.0000
Central nervous system	3 (2.9)	1 (2.4)	2 (3.6)	1.0000
Skin and soft tissue	4 (3.9)	0 (0.0)	4 (7.3)	**0.1332**
Bones and joints	1 (1.0)	1 (2.4)	0 (0.0)	0.4271
Cardiovascular	3 (2.9)	2 (4.9)	1 (1.8)	0.5739
Other	8 (7.8)	0 (0.0)	8 (14.5)	**0.0099**
Open fracture	4 (3.9)^g^	0 (0.0)	4 (7.3)^g^	**0.1332**
Late-onset neonatal bacterial infection	1 (1.0)^g^	0 (0.0)	1 (1.8)^g^	1.0000
Treatment used during the first bacterial infection episode and related to bacterial infection, n (%)				
Mechanical ventilation	57 (55.9)	25 (61.0)	28 (50.9)	0.3265
Non-invasive mechanical ventilation	20 (19.6)	11 (26.8)	8 (14.5)	**0.1351**
Invasive mechanical ventilation	37 (36.3)	14 (34.1)	20 (36.4)	0.8222
Inotropic or vasopressor support	17 (16.7)	6 (14.6)	8 (14.5)	0.9903
ECMO	1 (1.0)	0 (0.0)	0 (0.0)	—
Renal replacement therapy	1 (1.0)	0 (0.0)	0 (0.0)	—
Infection treated by surgery or percutaneous drainage	13 (12.7)	5 (12.2)	7 (12.7)	0.9378
Catheter removed and occurrence of apyrexia during the first 72 h of antibiotic treatment	12 (11.8)	4 (9.8)	7 (12.7)	0.7537

Data are number of episodes (%). P values were determined by χ² tests or Fisher’s exact tests

ARDS: Acute Respiratory Distress Syndrome; ECMO: Extracorporeal Membrane Oxygenation; HAP: Hospital-Acquired Pneumonia; VAP: Ventilator-Associated Pneumonia

^a^Six bacterial infection episodes were not assessable because of death. ^b^Four bacterial infection episodes were community and nosocomial. ^c^One bacterial infection episode was community and nosocomial. ^d^Two bacterial infection episodes were community and nosocomial. ^e^According to the 2005 International Pediatric Sepsis Consensus Conference. ^f^60 respiratory infection sites were ultimately retained in 59 episodes. ^g^11 other infection sites were ultimately retained in 8 episodes

Documented BIs concerned 49 first confirmed BI episodes. Microbiological data are presented in Table 3. The most prevalent causative bacteria encountered were *Staphylococcus* sp (34.7%), *Enterococcus* sp (16.3%) and *Klebsiella* sp (16.3%). Coagulase-negative *Staphylococci* (22.4%) were mostly methicillin-resistant (90.9% of the 11 episodes where coagulase-negative *Staphylococci* were isolated) while *Staphylococci aureus* (14.3%) were predominantly methicillin-sensitive (85.7% of the 7 episodes where *Staphylococcus aureus* was isolated). Extended-spectrum β-lactamase (ESBL)-producing Enterobacteriaceae were involved in only one of the 17 episodes in which Enterobacteriaceae were identified and no carbapenem-resistant pathogens were isolated.

**Table 3:** Microbiological data for first episodes of documented bacterial infection (*n* = 49)

Characteristics	*n* (%)
Sample site	
Bacteraemia	19 (38.8)
Intravascular catheter	3 (6.1)
Ear-Nose-Throat	1 (2.0)
Respiratory	18 (36.7)
Pleural fluid	2 (4.1)
Stools	1 (2.0)
Peritoneum	5 (10.2)
Ascites fluid	1 (2.0)
Urine	7 (14.3)
Cerebrospinal fluid	1 (2.0)
Skin and soft tissue	1 (2.0)
Bone	1 (2.0)
Mediastinum	1 (2.0)
Other	2 (4.1)
Number of causative bacteria	
One	32 (65.3)
Several	17 (34.7)
Causative bacteria	
Gram-positive bacteria	
* Staphylococcus* sp	17 (34.7)
* Staphylococcus aureus*	7 (14.3)
MSSA	6 (12.2)
MRSA	1 (2.0)
Coagulase-negative *Staphylococci*	11 (22.4)
Methicillin-resistant coagulase-negative *Staphylococci*	10 (20.4)
* Streptococcus* sp	6 (12.2)
* Streptococcus pneumoniae*	4 (8.2)
* Streptococcus agalactiae* (groupe B)	1 (2.0)
* Streptococcus mitis/oralis*	1 (2.0)
* Enterococcus* sp	8 (16.3)
* Turicella otitidis*	1 (2.0)
* Rothia mucilaginosa*	2 (4.1)
* Corynebacterium* sp	1 (2.0)
* Listeria monocytogenes*	1 (2.0)
Gram-negative bacteria	
Enterobacteriaceae	
* Escherichia coli*	5 (10.2)
* Klebsiella* sp	8 (16.3)
* Enterobacter* sp	7 (14.3)
* Serratia marcescens*	2 (4.1)
* Morganella morganii*	1 (2.0)
ESBL Enterobacteriaceae	1 (2.0)
Non-fermenting Gram-negative bacilli	
* Pseudomonas aeruginosa*	5 (10.2)
* Haemophilus influenzae*	4 (8.2)
* Haemophilus parinfluenzae*	1 (2.0)
* Branhamella catarrhalis*	1 (2.0)
* Campylobacter jejuni*	1 (2.0)
* Chryseobacterium*	2 (4.1)
* Leptospira* sp	1 (2.0)
Anaerobic bacteria	1 (2.0)

Data are number of episodes (%)

ESBL: Extended-Spectrum β-Lactamase producing; MSSA: Methicillin-Susceptible *Staphylococcus aureus*; MRSA: Methicillin-Resistant *Staphylococcus aureus*

### Non-compliance with antibiotic recommendations

As a result of death occurring during antibiotic treatment for 6 episodes (antibiotic therapy not completed), compliance for all parameters combined could only be analysed for 133 episodes. Non-compliance with recommendations for all parameters occurred in 51.1% of cases. Results of non-compliance with recommendations for each parameter are detailed in Table 4. The main reasons for non-compliance were inappropriate choice of antimicrobials (27.3%), duration of each antimicrobial treatment (26.3%) and length of antibiotic therapy (18.0%). In most cases, this duration was longer than that recommended by infection guidelines.

**Table 4:** Non-compliance with antibiotic recommendations for first suspected (or proven) bacterial infection episodes: type and source of non-compliance

Type and source of non-compliance	Number of episodes (%)	Number of error days, median (IQR)
Type of non-compliance		
Recommendations for all parameters (*n* = 133)	68 (51.1)	
Recommendations for length of antibiotic therapy (*n* = 133)	24 (18.0)	4 (2–6)
Prolonged duration	21	3 (2–6)
Insufficient duration	3	7 (7–10)
Recommendations for duration of each antimicrobial (*n* = 133)	35 (26.3)	3 (2–6)
Prolonged duration	30	3 (1–5)
Insufficient duration	5	6 (3–7)
Recommendations for choice of antimicrobials (*n* = 139)	38 (27.3)	
Spectrum too broad	19^a^	
Spectrum too narrow	16^a^	
Wrong spectrum of coverage entirely	6^a^	
Recommendations for number of antibiotic doses in 24 h (*n* = 139)	2 (1.4)	
Recommendations for daily dose of antibiotic therapy (*n* = 139)	21 (15.1)	
Recommendations for reassessment of antibiotic therapy at 72 h (*n* = 137)	22 (16.1)	
Source of non-compliance for all parameters	(*n* = 68)	
ICU	57 (83.8)^b^	
General paediatric ward	12 (17.6)^b^	

ICU: Intensive Care Unit

^a^3 episodes had two categories of non-compliance for choice of antimicrobials choice according to the initially suspected bacterial infection site and the bacterial infection site ultimately identified: one episode used antimicrobials with too broad a spectrum and the wrong spectrum of coverage entirely, another used antimicrobials with too broad a spectrum and too narrow a spectrum, and one episode used antimicrobials with spectrum too narrow a spectrum and the wrong spectrum of coverage entirely. ^b^For one episode, the error involved the ICU and the general paediatric ward

### Factors associated with Non-compliance with antibiotic recommendations

Results of univariate analyses to determine the significant factors associated with non-compliance with recommendations for all parameters are presented in Table 1 for all first suspected (or proven) BI episodes and in Table 2 for only first confirmed (documented or not) BI episodes. In multivariate analyses (Tables 5 and 6), independent risk factors of non-compliance for all parameters were the use of 2 or more antimicrobials per episode (OR 4.06, 95% CI 1.69–9.74, *p* = 0.0017) and the duration of broad-spectrum antibiotic therapy based on the standard definition of 4 days or more (OR 2.59, 95% CI 1.16–5.78, *p* = 0.0199) while independent protective factors of non-compliance (i.e., factors increasing compliance) for all parameters were respiratory failure as the reason for ICU admission (OR 0.36, 95% CI 0.14–0.90, *p* = 0.0281), using department protocol for antibiotic duration (OR 0.42, 95% CI 0.19–0.92, *p* = 0.0313) and aspiration pneumonia as BI site ultimately identified (OR 0.37, 95% CI 0.14–0.99, *p* = 0.0486).

**Table 5:** Significant independent factors for non-compliance with antibiotic recommendations for first suspected (or proven) bacterial infection episodes: results of multivariate analyses

Population type, non-compliance type and factors	Episodes in children with compliance with recommendations	Episodes in children with non-compliance with recommendations	Multivariate analysis	
			OR^a^ (95% CI)	*p* value
**Recommendations for all parameters** (*n* = 133)	(*n* = 65)	(*n* = 68)		
Reason for admission to ICU				
Respiratory failure	25 (38.5%)	10 (14.7%)	0.36 (0.14–0.90)	0.0281
Number of antimicrobials used per episode ≥ 2	35 (53.8%)	57 (83.8%)	4.06 (1.69–9.74)	0.0017
Duration of broad-spectrum antibiotic therapy, based on the standard definition ≥ 4 days	30 (46.2%)	48 (70.6%)	2.59 (1.16–5.78)	0.0199
Department protocol for antibiotic duration	35 (53.8%)	19 (27.9%)	0.42 (0.19–0.92)	0.0313
**Recommendations for length of antibiotic therapy** (*n* = 133)	(*n* = 109)	(*n* = 24)		
Reason for admission to ICU				
Neurologic compromise	11 (10.1%)	6 (25.0%)	3.41 (1.04–11.20)	0.0431
Initially suspected (or proven) bacterial infection site(s) for the first suspected (or proven) bacterial infection episode				
Catheter-related bacteraemia	18 (16.5%)	9 (37.5%)	3.70 (1.32–10.40)	0.0130
Duration of broad-spectrum antibiotic therapy, based on the standard definition ≥ 4 days	60 (55.0%)	18 (75.0%)	2.86 (1.00-8.15)	0.0499
**Recommendations for duration of each antimicrobial treatment** (*n* = 133)	(*n* = 98)	(*n* = 35)		
Initially suspected (or proven) bacterial infection site(s) for the first suspected (or proven) bacterial infection episode				
Catheter-related bacteraemia	14 (14.3%)	13 (37.1%)	5.42 (1.95–15.07)	0.0012
Other	15 (15.3%)^b^	10 (28.6%)^b^	3.29 (1.16–9.34)	0.0252
Duration of broad-spectrum antibiotic therapy, based on the standard definition ≥ 4 days	50 (51.0%)	28 (80.0%)	5.59 (2.01–15.60)	0.0010
**Recommendations for choice of antimicrobials** (*n* = 139)	(*n* = 101)	(*n* = 38)		
≥ 1 risk factor for ESBL Enterobacteriaceae^c^	23 (22.8%)	18 (47.4%)	2.56 (1.07–6.14)	0.0353
Patient context for onset of first suspected (or proven) bacterial infection episode				
Respiratory failure	26 (25.7%)	2 (5.3%)	0.18 (0.04–0.85)	0.0300
Number of antimicrobials used per episode ≥ 3	30 (29.7%)	21 (55.3%)	2.98 (1.21–7.32)	0.0173
**Recommendations for daily dose of antibiotic therapy** (*n* = 139)	(*n* = 118)	(*n* = 21)		
Initially suspected (or proven) bacterial infection site(s) for the first suspected (or proven) bacterial infection episode				
Respiratory	70 (59.3%)	8 (38.1%)	0.28 (0.09–0.83)	0.0223
**Recommendations for reassessment of antibiotic therapy at 72 h** (*n* = 137)	(*n* = 115)	(*n* = 22)		
Duration of broad-spectrum antibiotic therapy, according to the AWaRe classification (Watch and Reserve antibiotics) ≥ 3 days	55 (47.8%)	18 (81.8%)	3.98 (1.23–12.95)	0.0216
Department protocol for antibiotic duration	49 (42.6%)	5 (22.7%)	0.21 (0.06–0.71)	0.0125

Data are number of episodes (%)

AWaRe: Access, Watch, Reserve; ESBL: Extended-Spectrum β-Lactamase producing; ICU: Intensive Care Unit; OR: Odds Ratio

^a^The multivariate ORs were adjusted for centre when necessary. ^b^Other infection sites included late-onset neonatal bacterial infection (*n* = 11), paediatric inflammatory multisystem syndrome temporally associated with SARS-CoV-2 infection (*n* = 3), open fracture (*n* = 4), deep surgical site infection not elsewhere classified (*n* = 2), dental trauma (*n* = 2), retroperitoneal abscess (*n* = 1), conjunctivitis (*n* = 1), and chondritis and parotitis abscess (*n* = 1). ^c^Risk factors for ESBL Enterobacteriaceae: antibiotic therapy with Amoxicillin-clavulanic acid, 2nd or 3rd generation cephalosporin, fluoroquinolone (including single dose) or Piperacillin-tazobactam in the past 3 months, travel in endemic ESBL Enterobacteriaceae areas within the previous 3 months, living in a long-term facility and having an indwelling catheter and/or gastrostomy

**Table 6:** Significant independent factors for non-compliance with antibiotic recommendations for first episodes of confirmed (documented or not) bacterial infection: results of multivariate analyses

Population type, non-compliance type and factors	Episodes in children with compliance with recommendations	Episodes in children with non-compliance with recommendations	Multivariate analysis	
			OR^a^ (95% CI)	*p* value
**Recommendations for all parameters** (*n* = 96)	(*n* = 41)	(*n* = 55)		
Bacterial infection site(s) ultimately identified for the first bacterial infection episode				
Aspiration pneumonia	13 (31.7%)	8 (14.5%)	0.37 (0.14–0.99)	0.0486
**Recommendations for length of antibiotic therapy** (*n* = 96)	(*n* = 77)	(*n* = 19)		
Number of acute organ dysfunctions related to the first bacterial infection episode^b^ (only for sepsis, severe sepsis and septic shock)	(*n* = 58)	(*n* = 12)		
≥1	44 (75.9%)	6 (50.0%)	0.19 (0.04–0.85)	0.0300
Bacterial infection site(s) ultimately identified for the first bacterial infection episode				
Other	4 (5.2%)	4 (21.1%)	15.88 (2.41-104.76)	0.0041
**Recommendations for duration of each antimicrobial treatment** (*n* = 96)	(*n* = 66)	(*n* = 30)		
Number of acute organ dysfunctions related to the first bacterial infection episode^b^ (only for sepsis, severe sepsis and septic shock)	(*n* = 49)	(*n* = 21)		
≥2	6 (12.2%)	7 (33.3%)	4.21 (1.42–12.55)	0.0098
**Recommendations for choice of antimicrobials** (*n* = 102)	(*n* = 70)	(*n* = 32)		
Bacterial infection site(s) ultimately identified for the first bacterial infection episode				
Late-onset VAP	2 (2.9%)	5 (15.6%)	6.30 (1.15–34.44)	0.0338
**Recommendations for daily dose of antibiotic therapy** (*n* = 102)	(*n* = 84)	(*n* = 18)		
Severity of the first bacterial infection episode^b^				
Sepsis, severe sepsis, or septic shock	67 (79.8%)	9 (50.0%)	0.25 (0.09–0.74)	0.0117
**Recommendations for reassessment of antibiotic therapy at 72 h** (*n* = 100)	(*n* = 83)	(*n* = 17)		
Bacterial infection site(s) ultimately identified for the first bacterial infection episode				
Non-ventilator HAP	4 (4.8%)	4 (23.5%)	6.08 (1.35–27.37)	0.0188

Data are number of episodes (%)

HAP: Hospital-Acquired Pneumonia; OR: Odds Ratio; VAP: Ventilator-Associated Pneumonia

^a^The multivariate ORs were adjusted for centre when necessary. ^b^According to the 2005 International Pediatric Sepsis Consensus Conference

Similarly, we performed univariate and multivariate analyses to establish the independent predictive factors of non-compliance with recommendations for each parameter. Results of multivariate analyses are set out in Tables 5 and 6. In addition to those mentioned above, other independent risk factors for non-compliance were: (1) neurologic compromise as the reason for ICU admission; (2) suspected catheter-related bacteraemia; (3) suspected or confirmed BI site classified as “other” (for which antibiotic therapy is often poorly defined by guidelines); (4) late-onset ventilator-associated pneumonia (VAP) or non-ventilator hospital-acquired pneumonia (HAP); (5) presence of ≥ 1 risk factor for ESBL Enterobacteriaceae; and (6) duration of broad-spectrum antibiotic therapy ≥ 3 days according to the AWaRe classification (Watch and Reserve antibiotics). For children with neurologic compromise at time of ICU admission, the length of antibiotic therapy was prolonged for all non-compliant episodes and concerned aspiration pneumonia in half of all cases, catheter-related bacteraemia and one episode of BI that was finally ruled out. For late-onset VAP, non-compliance regarding the choice of antimicrobials involved empiric therapy for all non-compliant episodes. Otherwise, the independent factors for compliance were respiratory failure at the onset of suspected BI and suspected respiratory BI site. Notably, patient severity during the first suspected (or proven) BI episode, evaluated by PELOD-2 and pSOFA scores, did not represent an independent factor for non-compliance with recommendations. Serious infection (sepsis, severe sepsis and septic shock), particularly with one or more organ dysfunctions, constituted an independent factor for compliance in terms of length and daily dose of antibiotic therapy while the presence of ≥ 2 organ dysfunctions related to infection was an independent risk factor for non-compliance regarding the duration of each antimicrobial treatment. For sepsis with ≥ 2 organ dysfunctions, the duration of one or more antimicrobials was prolonged for all non-compliant episodes. This extended duration concerned antimicrobials used for VAP and non-ventilator HAP for 3 episodes and catheter-related bacteraemia, primary bacteraemia, pneumonia with parapneumonic pleural effusion, BI site classified as “other” and a final diagnosis of no BI for one episode each.

Finally, the use of an ASP during a suspected (or proven) BI episode did not modify the effect of independent variables on non-compliance with recommendations for all parameters (Table 7) and for each compliance parameter (results not shown). Indeed, all 95% CIs for stratified ORs overlapped in the two sub-groups although some independent variables were significant only in the ASP sub-group.

**Table 7:** Antimicrobial stewardship programme-stratified univariate analysis: significant factors associated with non-compliance with antibiotic recommendations for all parameters and comparison with all first suspected (or proven) bacterial infection episodes

	Episodes with no ASP (*n* = 73)		Episodes with ASP (*n* = 60)		All Episodes (*n* = 133)	
	OR (95% CI)	p value	OR (95% CI)	p value	OR (95% CI)	p value
Reason for admission to ICU						
Respiratory failure	0.30 (0.09–0.88)	**0.034**	0.24 (0.06–0.83)	**0.031**	0.28 (0.12–0.62)	**0.003**
Patient context for onset of first suspected (or proven) bacterial infection episode						
Respiratory failure	0.31 (0.09–0.96)	0.051	0.15 (0.02–0.67)	**0.025**	0.24 (0.09–0.59)	**0.003**
Initially suspected (or proven) bacterial infection site(s)						
Aspiration pneumonia	0.97 (0.31–2.97)	0.95	0.18 (0.03–0.81)	**0.042**	0.53 (0.21–1.25)	0.15
Final diagnosis						
No bacterial infection	0.38 (0.14–0.98)	**0.048**	0.33 (0.04–1.69)	0.21	0.40 (0.18–0.88)	**0.024**
Number of antimicrobials used per episode ≥ 2	4.24 (1.62–11.8)	**0.004**	11.4 (1.89–220)	**0.027**	4.44 (2.03–10.3)	**< 0.001**
Duration of broad-spectrum antibiotic therapy, based on the standard definition ≥ 4 days	2.91 (1.14–7.71)	**0.028**	2.94 (0.95–9.91)	0.068	2.80 (1.38–5.80)	**0.005**
Duration of broad-spectrum antibiotic therapy, according to the AWaRe classification (Watch and Reserve antibiotics) ≥ 3 days	9.09 (3.07–31.6)	**< 0.001**	1.54 (0.49–5.04)	0.46	3.35 (1.66–6.92)	**< 0.001**
Department protocol						
for antibiotic duration	0.34 (0.12–0.94)	**0.041**	0.29 (0.10–0.82)	**0.022**	0.33 (0.16–0.68)	**0.003**
for the choice of antimicrobials	0.40 (0.14–1.07)	0.073	0.28 (0.09–0.81)	**0.021**	0.36 (0.17–0.73)	**0.005**

ASP: Antimicrobial Stewardship Programme; AWaRe: Access, Watch, Reserve; ICU: Intensive Care Unit; OR: Odds Ratio

## Discussion

In this prospective observational multicentre study, we assessed compliance with recommendations for antibiotic prescriptions made by intensivists in a large population of children hospitalised in 8 French ICUs, most of which have an ASP (once weekly infection multidisciplinary staff meeting with audit and feedback). We also analysed the factors associated with non-compliance. Half of the prescriptions complied with guidelines. In the cases where recommendations were not followed, the main reasons for non-compliance were inappropriate choice of antimicrobial(s), inappropriate duration of one or more antimicrobials and inappropriate length of antibiotic therapy (most frequently prolonged duration for both). In multivariate analyses, we identified situations where intensivists were more attentive to recommendations: patients with respiratory failure, when a respiratory site was initially suspected, when aspiration pneumonia was the ultimately identified site and when antibiotic protocols were available in their ICU. Conversely, we highlighted contexts where risk of non-compliance with guidelines is likely to exist: prescribing at least two antibiotics, duration of broad-spectrum antibiotic therapy ≥ 3–4 days, neurologic compromise at time of ICU admission, suspicion of catheter-related bacteraemia, suspecting or confirming a BI site for which antibiotic therapy is often poorly defined by guidelines (classified as “other”), late-onset VAP, non-ventilator HAP and presence of ≥ 1 risk factor for ESBL Enterobacteriaceae. In sepsis patients, the presence of ≥ 2 organ dysfunctions related to infection represented an independent factor of non-compliance for duration of each antimicrobial treatment while one or more organ dysfunctions were an independent factor of compliance for length of antibiotic therapy.

A few studies have looked at antibiotic prescribing in paediatric ICUs. A prospective multicentre paediatric study of ventilator-associated lung disease showed 70% compliance before implementation of a local protocol and 76% afterwards [37]. Another study described inappropriate antibiotic prescribing in paediatric ICUs ranging from 16.7 to 61.9% depending on the evaluator and the period [38]. In ICU adults with sepsis, studies have demonstrated a comparable compliance rate between 47% and 58% [39, 40].

Lindberg et al. reported that non-compliance was independently associated with an increased risk of 30-day mortality corresponding to 1.86 (95% CI 1.34 to 2.58, *p* < 0.001) for partial compliance and 2.18 (95% CI 1.34 to 3.40, *p* < 0.001) for complete non-compliance [40]. In our study, we did not find a statistically significant difference in all-cause mortality between the compliant and non-compliant episodes (7.7% versus 5.9% respectively). However, mortality in our population was 10.8%, much lower than in critically ill adults and our study was not designed to evaluate the impact of non-compliance on mortality. Similarly, non-compliance was not associated with recurrence of bacterial infection in our work although the recurrence rate was only 1.4%.

Good compliance with recommendations for respiratory patients is consistent with clinical practice. Indeed, respiratory infections were mainly community-acquired in our study. Antibiotic therapy for these infections is well-defined by current guidelines for children [5, 6, 9] which allows doctors to better comply with recommendations.

The advantage of antibiotic protocols has already been identified. Protocols, based on current guidelines and updated regularly, are available 24/7 and enable harmonisation of prescriptions made by intensivists that can change from day to day. In adults, the implementation of computerised local antibiotic therapy protocols has been associated with reduced antibiotic exposure and mortality in adult ICUs [41].

To steer decisions about antibiotic therapy in ICUs, a European expert panel has created an “antibiotic care bundle” (ABC-Bundle) with evidence-based recommendations for antibiotic prescribing [42]. The six steps are: (1) provide rationale for antibiotic start; (2) perform appropriate microbiological sampling; (3) prescribe empiric antibiotic therapy according to guidelines (day 1); (4) review diagnosis; (5) assess de-escalation based on microbiological results (days 2–5); and (6) consider discontinuation of treatment in patients with negative culture results and clinical improvement (days 3–5). To mitigate the risk of antibiotic resistance emergence, recent European recommendations have defined antimicrobial de-escalation (ADE) as discontinuing one or more antimicrobials in the empiric combination therapy or replacing broad-spectrum antimicrobials with narrower spectrum agents [43]. In our study, ADE was evaluated by the duration of each antimicrobial treatment, reassessment of antibiotic therapy at 72 h and choice of antimicrobials (narrow antimicrobial therapy once pathogen identification and susceptibility testing results are available). Based on the DIANA study [44], we decided that ADE should take place within the first 3 days of initiation of empiric therapy to be appropriate. In the DIANA study in which 152 adult ICUs in 28 countries participated, ADE within the first 3 days of empiric therapy occurred in only 16% of patients while combination therapy was prescribed in half of the patients and infections were documented in 56% of the study population. Our results concerning inappropriate choice of antimicrobials and duration of one or more antimicrobials as the most frequent reasons for non-compliance and the number of antimicrobials used per episode and length of broad-spectrum antibiotic therapy to be representative of independent risk factors for non-compliance, show that ADE is essential in clinical practice. Recent recommendations on sepsis management in children have also emphasised that dual therapy is no longer recommended for children who are not immunocompromised and present no risk of carrying multidrug-resistant bacteria [4].

ADE is more frequently used in patients with a favourable course [45]. To analyse compliance with antibiotic recommendations, our paediatric infectious disease experts took into account patient improvement. Despite a favourable trajectory, ADE was often missing in patients with the most serious infections (sepsis with ≥ 2 organ dysfunctions). We hypothesise that intensivists probably delayed or did not achieve de-escalation due to the initial severity of the infection. However, de-escalation is safe for patients with favourable evolution. De Bus and colleagues reported no significant difference in 28-day mortality and infection relapse for ADE patients versus non-ADE patients in adult ICUs [44]. The 2021 SSC adult guidelines also suggested daily assessment for ADE and early ADE based on adequate clinical improvement for adults with sepsis or septic shock [3].

In patients with neurologic compromise at time of ICU admission, non-compliance in terms of length of antibiotic therapy was due to aspiration pneumonia in 50% of the patients while aspiration pneumonia represented a factor exhibiting good compliance for the entire study population. We assume that intensivists considered these patients to be at higher risk. A recent study, including 27,455 hospitalised children with neurologic impairment and pneumonia, showed that there were more systemic complications (acute respiratory failure, sepsis or ECMO) in neurologically impaired children with aspiration pneumonia [46].

Early administration of appropriate empiric therapy in patients with sepsis is crucial to reduce mortality [3]. In children with sepsis-associated organ dysfunction and septic shock, the 2020 SSC paediatric guidelines recommended empiric broad-spectrum therapy with one or more antimicrobials to cover all likely pathogens, taking into account local epidemiology, patient characteristics (age, patient history, allergies, MDR status) and suspected BI sites [4]. We found that late-onset VAP was an independent factor for non-compliance regarding the choice of antimicrobials and empiric therapy was inappropriate for all non-compliant episodes. Furthermore, Mangino and colleagues reported a low rate of appropriate empiric therapy (44%) for HAP in adult ICUs despite the implementation of multimodal educational activities to teach ICU staff about the guidelines. The presence of ≥ 1 risk factor for ESBL Enterobacteriaceae was another aetiology for inappropriate empiric therapy in our study. However, local microbiological data from participating ICUs showed a low rate of ESBL pathogens and ESBL Enterobacteriaceae was isolated in only 2.0% of BI episodes while children presented one or more risk factors for ESBL Enterobacteriaceae in 29.5% of episodes. Indications of probabilistic antibiotic therapy covering ESBL Enterobacteriaceae are mainly based on adult recommendations [10, 20] and should perhaps be adapted to paediatrics.

In French Paediatric and Neonatal ICUs, antibiotic prescriptions are issued by intensivists. They have access to a possible audit with an infectious disease specialist over the telephone (at their discretion) on weekdays and sometimes during weekends as well as an ASP (multidisciplinary staff meeting with intensivists, microbiologists and paediatric infectious disease specialists) occurring once a week at most. Previous studies have reported that ASPs decreased inappropriate prescriptions, antibiotic consumption and drug resistance [47, 48]. We could not demonstrate better compliance with the use of an ASP. In stratified analyses, the use of an ASP occurring once a week at most did not modify the effect of independent variables on non-compliance with recommendations. However, a once weekly ASP does not allow for recommendations to be given to all patients on antibiotics or for the daily “correction” of non-compliance concerning the duration of antimicrobials or re-evaluation at 72 h. Moreover, we only identified the presence or absence of non-compliance without quantifying the number of days for each instance of non-compliance on a daily basis (except for duration of antibiotics).

This study has several limitations. Firstly, we arbitrarily chose several separate weeks to include patients and not just one study period. This is explained by the very time-consuming nature of data collection and by the lack of funding for this study. These separate periods enabled each participating centre to organise their data collection. As with point prevalence studies, such data collection can potentially be a source of selection bias and could limit the generalisation of our results to the entire paediatric ICU population. However, consecutive patients were included during the study periods and since these weeks were spread over one year, we were able to take into account seasonal variations specific to paediatrics. Furthermore, at the time of the study, three centres did not have an ASP but all centres had access to audits with an infectious disease specialist over the telephone on weekdays and sometimes during weekends. For centres implementing an ASP, there was a once weekly multidisciplinary staff meeting, with advice given for antibiotic prescription. Finally, local ICU microbiology data from the two years prior to the study period were missing for three participating centres, which may have impacted analysis of compliance regarding the choice of antimicrobials by the paediatric infectious disease expert. However, the low MDR rate for the episodes included and in the local epidemiology of the other centres suggest a minimal influence.

## Conclusions

In French Paediatric and Neonatal ICUs, most of which hold a once weekly ASP, half of antibiotic prescriptions remain non-compliant with guidelines. For respiratory illnesses with clear treatment guidelines or for which antibiotic protocols already exist at a given centre, antibiotic use by intensivists tended to be more compliant. This highlights the importance of developing consensus/guidelines about treating specific illnesses and antibiotic protocols based on current guidelines that are updated regularly for use by clinicians. The benefit of using several antimicrobials and broad-spectrum antibiotic therapy should be reassessed daily by intensivists. Based on microbiological results and patient evolution, intensivists must perform an early de-escalation and stop antibiotics that are no longer indicated. A daily ASP could improve compliance with guidelines in these non-compliance prone situations.

### Electronic supplementary material

Below is the link to the electronic supplementary material.

Supplementary Material 1

## Data Availability

All data generated or analysed during this study are included in this published article and its supplementary information files.

## Abbreviations

ADE: Antimicrobial De-Escalation
ASP: Antimicrobial Stewardship Programme
BI: Bacterial Infection
CI: Confidence Interval
ESBL: Extended-Spectrum β-Lactamase producing
ESCMID: European Society of Clinical Microbiology and Infectious Diseases
GFRUP: Groupe Francophone de Réanimation et d’Urgences Pédiatriques (French-Speaking Group for Paediatric Intensive and Emergency Care)
GPIP: Groupe de Pathologies Infectieuses en Pédiatrie (French Group for Paediatric Infectious Diseases)
HAP: Hospital-Acquired Pneumonia
HAS: Haute Autorité de Santé (French National Authority for Health)
ICU: Intensive Care Unit
IDSA: Infectious Diseases Society of America
IPSC: International Pediatric Sepsis Consensus Conference
IQR: Inter-Quartile Range
MDR: Multidrug-Resistant
OR: Odds Ratio
PELOD-2: Pediatric Logistic Organ Dysfunction-2
pSOFA: Pediatric Sequential Organ Failure Assessment
SFAR: Société Française d’Anesthésie Réanimation (French Society of Anaesthesia and Intensive Care Medicine)
SPILF: Société de Pathologie Infectieuse de Langue Française (French Language Society for Infectious Diseases)
SRLF: Société de Réanimation de Langue Française (French Language Society for Intensive Care Medicine)
SSC: Surviving Sepsis Campaign
VAP: Ventilator-Associated Pneumonia
