# Supplementary material for: Compliance with antibiotic therapy guidelines in french paediatric intensive care units: a multicentre observational study
Source: BMC Infect Dis. 2024 Jun 12;24:582. doi: 10.1186/s12879-024-09472-0 (PMC11170905; doi:10.1186/s12879-024-09472-0)
Supplement: Supplementary file 1 — Supplementary Material 1 [file 12879_2024_9472_MOESM1_ESM.docx]

**Supplementary Appendix to:**

**Compliance with Antibiotic Therapy Guidelines in French Paediatric Intensive Care Units: A Multicentre Observational Study**

Romain Amadieu^1^, MD, Camille Brehin^2,3^, MD, PhD, Adéla Chahine^1^, MD, Erick Grouteau^2,3^, MD, Damien Dubois^4^, MD, PhD, Caroline Munzer^5^, Clara Flumian^5^, Olivier Brissaud^6^, MD, PhD, Barbara Ros^6^, MD, Gael Jean^6^, MD, Camille Brotelande^7^, MD, Brendan Travert^8^, MD, Nadia Savy^9^, MD, Benoit Bœuf^9^, MD, Ghida Ghostine^10^, MD, Isabelle Popov^10^, Pauline Duport^11^, MD, Richard Wolff^12^, MD, Laure Maurice^12^, MD, Stephane Dauger^12^, MD, PhD and Sophie Breinig^1^, MD

^1^ Neonatal and Paediatric Intensive Care Unit, Children’s Hospital, Toulouse University Hospital, Toulouse, France.

^2^ Paediatric Infectious Diseases Department, Children’s Hospital, Toulouse University Hospital, Toulouse, France.

^3^ General Paediatrics Department, Children’s Hospital, Toulouse University Hospital, Toulouse, France.

^4^ Bacteriology-Hygiene Department, Toulouse University Hospital, Toulouse, France.

^5^ Paediatric Clinical Research Department, Equipe MéDatAS-CIC 1436, Children's Hospital, Toulouse University Hospital, Toulouse, France.

^6^ Neonatal and Paediatric Intensive Care Unit, Pellegrin University Hospital, Bordeaux University, Bordeaux, France.

^7^ Paediatric Intensive Care Unit, Arnaud de Villeneuve University Hospital, Montpellier University, Montpellier, France.

^8^ Neonatal and Paediatric Intensive Care Unit, Mère-Enfant University Hospital, Nantes University, Nantes, France.

^9^ Neonatal and Paediatric Intensive Care Unit, Estaing University Hospital, Clermont-Ferrand University, Clermont-Ferrand, France.

^10^ Neonatal and Paediatric Intensive Care Unit, Amiens-Picardie University Hospital, Amiens University, Amiens, France.

^11^ Neonatal and Paediatric Intensive Care Unit, Felix Guyon University Hospital, La Réunion University, Saint-Denis, Ile de la Réunion, France.

^12^ Paediatric Intensive Care Unit, Assistance Publique-Hôpitaux de Paris, Robert Debré University Hospital, Paris University, Paris, France.

**Table of Contents**

| **Appendix 1** | **Supplementary Table 1**. Characteristics of the eight participating intensive care units. | p. 3 |
| --- | --- | --- |
| **Appendix 2** | **Supplementary Methods** | p. 4 |
| **Appendix 3** | **Supplementary Table 2**. Duration of antibiotic therapy as recommended by guidelines used for analysis of compliance by paediatric infectious disease experts. | p. 7 |
|  | **Supplementary Table 3**. Choice of antimicrobials, number of antibiotic doses in 24 hours and daily dose of antibiotic therapy as recommended by guidelines used for analysis of compliance by paediatric infectious disease experts. | p. 11 |
| **Appendix 4** | **Local ICU Microbiological Data** | p. 32 |

# Appendix 1.

**Supplementary Table 1**. Characteristics of the eight participating intensive care units.

|  | Centres | | | | | | | |
| --- | --- | --- | --- | --- | --- | --- | --- | --- |
|  | 1 | 2 | 3 | 4 | 5 | 6 | 7 | 8 |
| City | Toulouse | Bordeaux | Montpellier | Nantes | Clermont-Ferrand | Amiens | Paris  Robert Debré | Saint-Denis Réunion |
| Beds | 22 | 16 | 8 | 12 | 6 | 16 | 20 | 12 |
| Annual admissions | 918 | 974 | 550 | 734 | 390 | 490 | 1150 | 516 |
| Patient population |  |  |  |  |  |  |  |  |
| Neonatal | Yes | Yes | Yes | Yes | Yes | Yes | No | Yes |
| Paediatric | Yes | Yes | Yes | Yes | Yes | Yes | Yes | Yes |
| Mixed medical and surgical | Yes | Yes | Yes | Yes | Yes | Yes | Yes | Yes |
| Cardiac surgery | Yes | No | No | Yes | No | No | No | Yes |
| Antimicrobial stewardship programme | Yes | Yes | No | Yes | No | No | Yes | Yes |
| Infectious multidisciplinary staff meeting^a^ | Yes | Yes | No | Yes | No | No | Yes | Yes |
| Frequency | Once a week | Once a week | ⸺ | Once a week | ⸺ | ⸺ | Once a week | Once every 10 days |
| Possible audit with infectious disease specialist by phone call | Yes | Yes | Yes | Yes | Yes | Yes | Yes | Yes |
| Weekdays | Yes | Yes | Yes | Yes | Yes | Yes | Yes | Yes |
| Weekends | Yes | No | Yes | No | Yes | No | No | No |

^a^Infectious multidisciplinary staff meeting included microbiologists, infectious diseases specialists and intensivists.

# Appendix 2.

**Supplementary Methods:**

## Data Collection

So long as patients received antibiotics in hospital for the first suspected or proven bacterial infection episode (ICU and general paediatric ward if antibiotic therapy was not completed in the ICU), the following data were prospectively collected on a daily basis and reported in a standardised Case Report Form (CRF):

1. Patient characteristics:

- Date of birth.
- Birth status (born full term or born prematurely).
- Post-menstrual age (if born prematurely).
- Weight.
- Sex.
- ICU admission data: date of admission to ICU, reason for ICU admission, Paediatric Index of Mortality-2 (PIM-2) score at ICU admission.
- Surgical history within 30 days before the onset of the first suspected or proven bacterial infection episode.
- Comorbidities according to the Paediatric Complex Chronic Conditions (CCC) classification system version 2 based on ICD-10 diagnostic coding.
- Vaccination status.
- Allergy to antibiotics.
- Multidrug-resistant (MDR) status: colonisation or previous infection, risk factor.
- Patient context for onset of first suspected or proven bacterial infection episode.

1. Characteristics of the first suspected or proven bacterial infection episode:

- Bacterial infection site(s) initially suspected.
- Final diagnosis −no bacterial infection, presumed [i.e., not documented] bacterial infection or documented bacterial infection−.
- Community or nosocomial origin of the infection.
- Infection severity according to the 2005 International Pediatric Sepsis Consensus Conference (IPSC).
- Acute organ dysfunctions related to the infection as defined by 2005 IPSC.
- Bacterial infection site(s) ultimately identified.
- Causative bacteria.

1. Organ dysfunction scores for the first suspected or proven bacterial infection episode: Pediatric Logistic Organ Dysfunction-2 (PELOD-2) and Pediatric Sequential Organ Failure Assessment (pSOFA) scores (value on Day 0 and maximal value during the first episode).
2. Antibiotic therapy for the first suspected or proven bacterial infection episode:

- Starting date, starting time, discontinuation date, discontinuation time, route of administration, dose per 24 hours and number of antibiotic doses in 24 hours for each antibiotic administered.
- Audit with an antimicrobial stewardship team.
- Department protocol.
- Situation where the duration of antibiotic therapy was longer than that recommended by infectious guidelines in the event of favourable evolution: slow clinical biological and/or radiological course, complication.

1. Concomitant therapeutics other than antibiotics used within 48 hours before the onset of the first suspected or proven bacterial infection episode and during the first suspected or proven bacterial infection episode:

- Mechanical ventilation.
- Vasoactive drugs (Dobutamine, Dopamine, Epinephrine, Norepinephrine, Milrinone).
- Extracorporeal membrane oxygenation (ECMO).
- Renal replacement therapy.
- Infection treated by surgery, percutaneous drainage or endoscopic drainage.
- Catheter removed and occurrence of apyrexia during the first 72 hours of antibiotic treatment.

## Definitions

- Surgical antimicrobial prophylaxis was defined as the use of antimicrobial agents immediately before the procedure, during the procedure and up to 48 hours afterwards to prevent surgical site infections.
- Neonates were defined as patients with chronological age at Day 0 (day of antibiotic therapy initiation) >72 hours and (i) ≤28 days if born full term (gestational age ≥37 weeks) or (ii) with post-menstrual age at Day 0 ≥37 weeks + 0 day and ≤44 weeks + 6 days if born prematurely (gestational age <37 weeks).
- Final diagnosis of the first suspected or proven bacterial infection episode was decided by the clinical team in charge of the child. Based on clinical signs as well as biological, radiological and microbiological results used in daily practice, each episode was classified into one of three following categories: no bacterial infection, presumed [i.e., not documented] bacterial infection or documented bacterial infection.
- The duration of antibiotic therapy for the first suspected or proven bacterial infection episode was the time interval expressed in days between the starting time of systemic (intravenous, intramuscular or oral) antibiotics (Day 0) and discontinuation time of the antibiotic therapy. If the patient was discharged from hospital before the end of antibiotic treatment, data related to outpatient antibiotic therapy given for the first bacterial infection episode were collected along with the discharge medical prescription.
- To identify broad-spectrum antibiotics, we used two separate definitions. Based on the standard definition, broad-spectrum antibiotics were all antibiotics except those with useful activity against only gram-positive bacteria (penicillin G, oxacillin, vancomycin, teicoplanin, linezolid, macrolides, clindamycin) and those whose predominant activity is acting against gram-positive bacteria (amoxicillin, 1st generation cephalosporin [cefazolin, cephalexin] as well as 2nd generation cephalosporin [cefuroxime, cefaclor, cefamandole], rifampicin). According to the 2019 AWaRe (Access, Watch, Reserve) classification based on the recommendation of a WHO Expert Committee, we considered broad-spectrum antibiotics to be those belonging to Watch and Reserve groups.

## Analysis of Compliance with Antibiotic Recommendations

Two paediatric infectious disease experts both analysed compliance with antibiotic recommendations for each first suspected or proven BI episode, taking into account the following parameters: length of antibiotic therapy, duration of each antimicrobial treatment, choice of antimicrobials, number of antibiotic doses in 24 hours, daily dose of antibiotic therapy and reassessment of antibiotic therapy at 72 hours.

The experts considered an episode to be compliant for the duration of antibiotic therapy if: 1. duration was that recommended by infectious guidelines (mainly the SPILF and GPIP French guidelines) by accepting longer durations in the case of slow clinical biological and/or radiological course or complications; and 2. all antimicrobials were discontinued within 72 hours of initiation of empiric therapy if bacterial infection was ruled out. In the event of documented infection, length of antibiotic therapy included the initial empiric antibiotic therapy when active against isolated bacteria.

The experts deemed an episode as compliant for the duration of each antimicrobial treatment if: 1. the duration of all antimicrobials used was that recommended by infectious guidelines (mainly the SPILF and GPIP French guidelines); 2. all antimicrobials were discontinued within 72 hours of initiation of empiric therapy if bacterial infection was ruled out; and 3. antimicrobials started for an initially suspected bacterial infection site were discontinued within 72 hours of initiation of empiric therapy if this bacterial infection site was finally ruled out (e.g., stopping vancomycin if catheter‑related infection was ultimately not confirmed). In the event of documented infection, the duration of antimicrobials included the initial empiric antibiotic therapy when active against isolated bacteria.

The experts felt an episode to be compliant for antimicrobial choice if: 1. the empiric therapy was that recommended by infectious guidelines (combination of French, European and American guidelines) according to local ICU ecology, patient characteristics (age, comorbidities, up-to-date vaccination status, allergy to antibiotics, MDR status) and bacterial infection characteristics (bacterial infection sites initially suspected, infection severity and causative bacteria); and 2. the empiric antimicrobial therapy was replaced by an agent with a narrower spectrum according to the causative pathogen identified, susceptibility testing results and bacterial infection sites ultimately identified.

Analyses of compliance for the number of antibiotic doses in 24 hours and daily dose of antibiotic therapy were based on a combination of French, European and American guidelines.

Finally, the experts considered an episode compliant for reassessment of antibiotic therapy at 72 hours if: 1. in the case of documented bacterial infection, the empiric antimicrobial therapy was narrowed according to the causative pathogen identified and susceptibility testing results; 2. all antimicrobials were discontinued within 72 hours of initiation of empiric therapy if bacterial infection was ruled out; and 3. antimicrobials started for an initially suspected bacterial infection site were stopped within 72 hours of initiation of empiric therapy if this bacterial infection site was finally ruled out (e.g., stopping vancomycin if catheter‑related infection was ultimately not confirmed).

# Appendix 3.

**Supplementary Table 2**. Duration of antibiotic therapy as recommended by guidelines used for analysis of compliance by paediatric infectious disease experts.

| **Bacterial infection site / bacteria** | **Antibiotics**^a^ | **Treatment duration in case of favourable outcome**^b^ | **Comments** |
| --- | --- | --- | --- |
| Uncomplicated primary bacteraemia (bacteraemia without identified portal of entry) (uncomplicated is defined by apyrexia and negative blood cultures occurring within 72 hours after initiation of antibiotic therapy, and by the absence of secondary localisation, infective endocarditis or infected thrombophlebitis) in a patient who has no intravascular device material (e.g., pacemaker or recently placed vascular graft) | | | |
| *Streptococcus pneumoniae* |  | 7 days |  |
| Other streptococci |  | 7 days |  |
| GNB (including enterobacteriaceae and non-fermenting GNB) |  | 7 days |  |
| Non-documented gut bacterial translocation |  | 7 days |  |
| Uncomplicated short-term central venous catheter-related bacteraemia (uncomplicated is defined by apyrexia and negative blood cultures occurring within 72 hours after initiation of antibiotic therapy, and by the absence of secondary localisation, infective endocarditis or infected thrombophlebitis) in a patient who has no intravascular device material (e.g., pacemaker or recently placed vascular graft) | | | |
| Coagulase-negative staphylococci and catheter removed |  | 3 days after catheter removal and initial negative blood culture |  |
| Coagulase-negative staphylococci and catheter retained |  | 10 days after initial negative blood culture | If no favourable course within 48 hours after initiation of appropriate antibiotic therapy (persistent bacteraemia or severe sepsis) or relapse, remove catheter and treat with systemic antibiotic therapy for 3 days after catheter removal and initial negative blood culture. |
| GNB and catheter removed |  | 7 days after catheter removal and initial negative blood culture |  |
| GNB and catheter retained |  | 10 days after initial negative blood culture | If no favourable course within 48 hours after initiation of appropriate antibiotic therapy (persistent bacteraemia or severe sepsis) or relapse, remove catheter and treat with systemic antibiotic therapy for 3 days after catheter removal and initial negative blood culture. |
| *Staphylococcus aureus* : **CATHETER REMOVAL** |  | 14 days after catheter removal and initial negative blood culture | Excluding: immunocompromised patients (hereditary immunodeficiency, neutropenia, HIV infection, immunosuppressive drugs); diabetic patients. |
| Ear-Nose-Throat |  |  |  |
| Non-severe cervical adenitis |  | 8 days |  |
| Severe cervical adenitis (signs of severity: severe sepsis or septic shock, compression, thrombosis) |  | 10 -15 days based on clinical and biological course |  |
| Purulent acute otitis media <2 years old |  | 10 days | For purulent acute otitis media with strongly suspected resistant pneumococcus and digestive intolerance: Ceftriaxone for 3 days. |
| Purulent acute otitis media ≥2 years old, without otorrhea or any relapse |  | 5 days | For purulent acute otitis media with strongly suspected resistant pneumococcus and digestive intolerance: Ceftriaxone for 3 days. |
| Purulent acute otitis media ≥2 years old, with otorrhea or recurrent otitis |  | 10 days | For purulent acute otitis media after failure of first treatment with Amoxicillin-clavulanic acid: Ceftriaxone 3 days. |
| Acute bacterial parotitis |  | 10 days |  |
| Respiratory |  |  |  |
| Community-acquired pneumonia | Amoxicillin or 3rd-generation cephalosporins | 5-7 days |  |
|  | or Pristinamycin | 10 days |  |
| Atypical community-acquired pneumonia | Clarithromycin or Josamycin or Pristinamycin | 10 days |  |
|  | or Azithromycin | 3 days |  |
| Aspiration pneumonia | Amoxicillin-clavulanic acid | 7 days | Alternative antibiotics: Ceftriaxone + Metronidazole for 5 days. |
| Hospital-acquired pneumonia (VAP or non-ventilator hospital-acquired pneumonia) |  | 7 days | Excluding: immunocompromised patients (hereditary immunodeficiency, neutropenia, HIV infection, immunosuppressive drugs); situations necessitating prolonged treatment (empyema, necrotising or abscessed pneumonia). |
| Uncomplicated parapneumonic pleural effusion (medical treatment without pleural drainage) |  | 10 days | Whether or not pleural puncturing occurs. |
| Community-acquired or healthcare-associated pleural empyema |  | 15 days starting with the date of installation of the last pleural drain |  |
| Intra-abdominal |  |  |  |
| Necrotising enterocolitis |  | 7-10 days based on clinical and biological course |  |
| Non-documented bacterial colitis |  | 3-5 days |  |
| Bacterial gastroenteritis due to *Campylobacter jejuni* | Azithromycin | 3 days |  |
|  | or Ciprofloxacin | 5 days |  |
| Typhoid fever without complications | Ceftriaxone | 5-7 days |  |
|  | or fluoroquinolone | 7 days |  |
| Localised community-acquired peritonitis |  | 3 days after surgery or drainage | If the infection source is controlled (surgery or percutaneous or endoscopic drainage) |
| Generalised community-acquired peritonitis |  | 4 days after surgery or drainage | If the infection source is controlled (surgery or percutaneous or endoscopic drainage). |
| Postoperative peritonitis |  | 8 days after surgery or drainage | If the infection source is controlled (surgery or percutaneous or endoscopic drainage). |
| Urinary tract |  |  |  |
| Uncomplicated acute cystitis in pubescent girls | Fosfomycin trometamol | A single dose |  |
|  | or Pivmecillinam | 3 days |  |
|  | or Nitrofurantoin | 3 days |  |
| Uncomplicated acute cystitis in prepubescent girls |  | 5 days |  |
| Catheter-associated acute cystitis |  | 3 days if symptoms improve following urinary catheter removal, 5-7 days in other cases |  |
| Acute cystitis with risk of complications (any urinary tract abnormality, uropathy, recent urologic procedure, pregnancy, creatinine clearance <30 ml/min/1.73m2, or immunodepression) | Trimethoprim-sulfamethoxazole | 5 days |  |
|  | or other antibiotics | 7 days | Fluroquinolones are contraindicated. |
| Acute pyelonephritis |  | 10 days (7 days if exclusively intravenous) |  |
| Hospital-acquired acute pyelonephritis with urinary tract drainage (except bladder catheterisation) |  | 10 days |  |
| Central nervous system |  |  |  |
| *Listeria monocytogenes* meningitis or gram-negative bacilli meningitis, except for *H. influenzae* meningitis |  | 21 days |  |
| Healthcare-associated meningitis caused by a coagulase-negative staphylococcus |  | 10-14 days after the last positive culture |  |
| Healthcare-associated meningitis caused *Staphylococcus aureus* |  | 10-14 days after the last positive culture |  |
| Skin and soft tissue |  |  |  |
| Trauma wound heavily soiled or on local ischaemic terrain, without or with localised lesions (erythema and induration extending < 5 cm from the wound edge, even if purulent lesion) and with no general signs |  | 5 days |  |
| Trauma wound with extended lesions (erythema and induration extending > 5 cm from the wound edge, lymphangitis) or general signs |  | 7 days |  |
| Superficial surgical site infection, with extended lesions |  | 7 days |  |
| Non-necrotising bacterial dermohypodermitis (cellulitis, erysipelas, caused by bites) |  | 7 days |  |
| Acute staphylococcus epidermolysis |  | 10 days |  |
| Pilonidal cyst infection |  | 15 days |  |
| Bones and joints |  |  |  |
| Healthcare-associated osteitis |  | 6 weeks |  |
| Cardiovascular |  |  |  |
| Postsurgical mediastinitis |  | 21 days |  |
| Myopericarditis due to *Leptospira* |  | 10-14 days |  |
| Bacteraemia secondary to an identified site of infection |  | Aside from the specific case of Staphylococcus aureus, duration of treatment for bacteraemia secondary to an identified site of infection corresponds to length of treatment for the source of bacteraemia. | |

GNB: Gram-Negative Bacilli; VAP: [Ventilator-Associated](https://context.reverso.net/traduction/anglais-francais/Ventilator-Associated) Pneumonia.

^a^Antibiotics are only mentioned when associated with a specific duration. ^b^In the case of documented infection, the length of antibiotic therapy includes the initial empiric antibiotic therapy when active against isolated bacteria.

**Supplementary Table 3**. Choice of antimicrobials, number of antibiotic doses in 24 hours and daily dose of antibiotic therapy as recommended by guidelines used for analysis of compliance by paediatric infectious disease experts.

| **Clinical situations or bacteria** | **Bacteriological target** | **Preferred antibiotics** | **Alternative antibiotics** | **Comments** |
| --- | --- | --- | --- | --- |
| Late-onset neonatal bacterial infection | | | | |
| Late-onset neonatal bacterial infection, age 3-7 days old, without neurological or circulatory failure signs, **empiric treatment** | *Streptococcus agalactiae* (Group B streptococcus), *Escherichia coli* | Cefotaxime IV 100 mg/kg/day in 2 divided doses |  |  |
|  |  | + Aminoglycoside for 48 hours: Gentamicin IV 5 mg/kg/day in 1 daily dose; or Amikacin IV 15 mg/kg/day in 1 daily dose |  |  |
| Late-onset neonatal bacterial infection, age 3-7 days old, with neurological or circulatory failure signs, **empiric treatment** | *Streptococcus agalactiae* (Group B streptococcus), *Escherichia coli*, *Listeria monocytogenes*, Enterococci | Cefotaxime IV 200 mg/kg/day in 2 divided doses |  |  |
|  |  | + Aminoglycoside for 48 hours: Gentamicin IV 5 mg/kg/day in 1 daily dose; or Amikacin IV 15 mg/kg/day in 1 daily dose |  |  |
|  |  | + Amoxicillin IV 200 mg/kg/day in 2 divided doses |  |  |
| Late-onset neonatal bacterial infection, age 8-28 days old, without neurological or circulatory failure signs, **empiric treatment** | *Streptococcus agalactiae* (Group B streptococcus), *Escherichia coli* | Cefotaxime IV 100 mg/kg/day in 3 divided doses |  |  |
|  |  | + Aminoglycoside for 48 hours: Gentamicin IV 5 mg/kg/day in 1 daily dose; or Amikacin IV 15 mg/kg/day in 1 daily dose |  |  |
| Late-onset neonatal bacterial infection, age 8-28 days old, with neurological or circulatory failure signs, **empiric treatment** | *Streptococcus agalactiae* (Group B streptococcus), *Escherichia coli*, *Listeria monocytogenes*, Entérococci | Cefotaxime IV 200 mg/kg/day in 3 divided doses |  |  |
|  |  | + Aminoglycoside for 48 hours: Gentamicin IV 5 mg/kg/day in 1 daily dose; or Amikacin IV 15 mg/kg/day in 1 daily dose |  |  |
|  |  | + Amoxicillin IV 200 mg/kg/day in 3 divided doses |  |  |
| Late-onset neonatal bacterial infection, age 1-3 months old, without neurological or circulatory failure signs, **empiric treatment** | *Streptococcus agalactiae* (Group B streptococcus), *Escherichia coli* | Cefotaxime IV 150 mg/kg/day in 3 divided doses |  |  |
|  |  | + Aminoglycoside for 48 hours: Gentamicin IV 5 mg/kg/day in 1 daily dose; or Amikacin IV 15 mg/kg/day in 1 daily dose |  |  |
| Late-onset neonatal bacterial infection, age 1-3 months old, with neurological or circulatory failure signs, **empiric treatment** | *Streptococcus agalactiae* (Group B streptococcus), *Escherichia coli* | Cefotaxime IV 200 mg/kg/day in 3 divided doses |  |  |
|  |  | + Aminoglycoside for 48 hours: Gentamicin IV 5 mg/kg/day in 1 daily dose; or Amikacin IV 15 mg/kg/day in 1 daily dose |  |  |
| Primary bacteraemia |  |  |  |  |
| Gut bacterial translocation, **empiric treatment** | Enterobacteriaceae | Cefotaxime IV 100 mg/kg/day (maximum 1 g q6-8h) in 3 or 4 divided doses; or Ceftriaxone IV 50 mg/kg/day (maximum 2 g/day) in 1 daily dose |  | In case of risk factors for ESBL Enterobacteriaceae, empiric therapy for gut bacterial translocation should include Piperacillin-tazobactam or a ß-lactam according to previous antibiotic susceptibility results, avoiding carbapenems as much as possible. |
|  |  | + Metronidazole IV 30 mg/kg/day (maximum 1.5 g/day) in 3 divided doses |  |  |
|  |  | +/- Aminoglycoside for at least 48 hours, if severe sepsis or septic shock: Amikacin IV 15-30 mg/kg/day in 1 daily dose; or Gentamicin IV 5-8 mg/kg/day in 1 daily dose |  |  |
| Primary bacteria due to Pneumococcus | *Streptococcus pneumoniae* | 3rd-generation cephalosporin: Cefotaxime IV 100 mg/kg/day (maximum 1 g q6-8h) in 3 or 4 divided doses; or Ceftriaxone IV 50 mg/kg/day (maximum 2 g/day) in 1 daily dose |  |  |
| Primary bacteria due to other Streptococci | Streptococci other than *Streptococcus pneumoniae* | 3rd-generation cephalosporin: Cefotaxime IV 100 mg/kg/day (maximum 1 g q6-8h) in 3 or 4 divided doses; or Ceftriaxone IV 50 mg/kg/day (maximum 2 g/day) in 1 daily dose |  |  |
| Catheter-related bacteremia |  |  |  |  |
| Central venous catheter-related bacteraemia, **empiric treatment** | Methicillin-resistant *Staphylococcus* | Vancomycin IV 40-60 mg/kg/day (maximum 4 g/day) in 4 divided doses or by continuous infusion (after loading dose of 15 mg/kg (maximum 2 g)) | In the presence of a high local MRSA prevalence with Vancomycin MIC >2 µg/ml, use Daptomycin | In addition to coverage for gram-positive pathogens (Vancomycin or Daptomycin), empiric therapy should include coverage for GNB if patients have a femoral catheter, if they have severe sepsis or septic shock, or if they are neutropenic: Ceftazidime, a 4th-generation cephalosporin (Cefepime), Piperacillin-tazobactam or a carbapenem. |
|  |  | +/- Gentamicin IV 5-8 mg/kg/day in 1 daily dose for at least 48 hours, if severe sepsis or septic shock | +/- Gentamicin IV 5-8 mg/kg/day in 1 daily dose for at least 48 hours, if severe sepsis or septic shock |  |
|  | + GNB (including enterobacteriaceae and non-fermenting GNB) | +/- Ceftazidime IV 100-200 mg/kg/day (maximum 6 g/day) in 4 divided doses, if severe sepsis or septic shock | +/- Aztreonam IV 120 mg/kg/day (maximum 6 g/day) in 4 divided doses, if severe sepsis or septic shock |  |
| Central venous catheter-related bacteraemia due to MSSA | MSSA | Cloxacillin IV 100-150 mg/kg/day (maximum 2 g q6h) in 4 divided doses | Vancomycin IV 40-60 mg/kg/day (maximum 4 g/day) in 4 divided doses or by continuous infusion (after loading dose of 15 mg/kg (maximum 2 g)) | Catheter removal.  Transesophageal echocardiography should be performed to rule out infective endocarditis.  Gentamicin combined with Cloxacillin or Cefazolin or Vancomycin or Teicoplanin. |
|  |  | or Cefazolin IV 150 mg/kg/day (maximum 6 g/day for <70 kg, 8-12 g/day for ≥70 kg) in 4 divided doses | or Teicoplanin IV or IM loading dose of 10 mg/kg 3 times 12 hours apart, then 6-10 mg/kg/day in 1 daily dose |  |
|  |  | +/- Gentamicin IV 5-8 mg/kg/day in 1 daily dose for at least 48 hours, if severe sepsis or septic shock | +/- Gentamicin IV 5-8 mg/kg/day in 1 daily dose for at least 48 hours, if severe sepsis or septic shock |  |
| Central venous catheter-related bacteraemia due to MRSA | MRSA | Vancomycin IV 40-60 mg/kg/day (maximum 4 g/day) in 4 divided doses or by continuous infusion (after loading dose of 15 mg/kg (maximum 2 g)) | In the case of Vancomycin MIC >2 µg/ml:  Daptomycin | Catheter removal.  Transesophageal echocardiography should be performed to rule out infective endocarditis.  Preferred antibiotics: Gentamicin in association with Vancomycin or Teicoplanin. |
|  |  | or Teicoplanin IV or IM loading dose of 10 mg/kg 3 times 12 hours apart, then 6-10 mg/kg/day in 1 daily dose | +/- Gentamicin IV 5-8 mg/kg/day in 1 daily dose for at least 48 hours, if severe sepsis or septic shock |  |
|  |  | +/- Gentamicin IV 5-8 mg/kg/day in 1 daily dose for at least 48 hours, if severe sepsis or septic shock |  |  |
| Central venous catheter-related bacteraemia due to Methicillin-susceptible coagulase-negative staphylococci | Methicillin-susceptible coagulase-negative staphylococci | Cloxacillin IV 100-150 mg/kg/day (maximum 2 g q6h) in 4 divided doses | In the case of allergy to penicillin:  Vancomycin IV 40-60 mg/kg/day (maximum 4 g/day) in 4 divided doses or by continuous infusion (after loading dose of 15 mg/kg (maximum 2 g)) | Gentamicin combined with Cloxacillin or Cefazolin or Vancomycin or Teicoplanin. |
|  |  | or Cefazolin IV 150 mg/kg/day (maximum 6 g/day for <70 kg, 8-12 g/day for ≥70 kg) in 4 divided doses |  |  |
|  |  | +/- Gentamicin IV 5-8 mg/kg/day in 1 daily dose for at least 48 hours, if severe sepsis or septic shock | or Teicoplanin IV or IM loading dose of 10 mg/kg 3 times 12 hours apart, then 6-10 mg/kg/day in 1 daily dose |  |
|  |  |  | +/- Gentamicin IV 5-8 mg/kg/day in 1 daily dose for at least 48 hours, if severe sepsis or septic shock |  |
| Central venous catheter-related bacteraemia due to Methicillin-resistant coagulase-negative staphylococci | Methicillin-resistant coagulase-negative staphylococci | Vancomycin IV 40-60 mg/kg/day (maximum 4 g/day) in 4 divided doses or by continuous infusion (after loading dose of 15 mg/kg (maximum 2 g)) |  | Gentamicin combined with Vancomycin or Teicoplanin. |
|  |  | or Teicoplanin IV or IM loading dose of 10 mg/kg 3 times 12 hours apart, then 6-10 mg/kg/day in 1 daily dose |  |  |
|  |  | +/- Gentamicin IV 5-8 mg/kg/day in 1 daily dose for at least 48 hours, if severe sepsis or septic shock |  |  |
| Central venous catheter-related bacteraemia due to *Serratia sp* | *Serratia sp* | Choose the antibiotic according to susceptibility testing results (fluoroquinolone or 4th-generation cephalosporin or Meropenem) |  | Catheter removal. |
|  |  | +/- Aminoglycoside for at least 48 hours, if severe sepsis or septic shock: Gentamicin IV 5-8 mg/kg/day in 1 daily dose; or Amikacin 15 mg/kg/day in 1 daily dose |  |  |
| Ear-Nose-Throat |  |  |  |  |
| Non-severe cervical adenitis, **empiric treatment** | *Streptococcus pyogenes* (Group A streptococcus), MSSA | Amoxicillin-clavulanic acid PO 80 mg/kg/day (maximum 2-3 g/day) in 2 or 3 divided doses (whatever the rapid detection test result) | Whatever the rapid detection test result: |  |
|  |  |  | Clarithromycin PO 15 mg/kg/day (maximum 500 mg/day) in 2 divided doses |  |
|  |  |  | or Josamycin PO 50 mg/kg/day (maximum 2 g/day) in 2 divided doses |  |
|  |  |  | or after 6 years: Clindamycin PO 30 mg/kg/day (maximum 1.8 g/day) in 3 divided doses |  |
| Severe cervical adenitis (signs of severity: severe sepsis or septic shock, compression, thrombosis), **empiric treatment** | *Streptococcus pyogenes* (Group A streptococcus), MSSA, *Streptococcus pneumoniae*, *Fusobacterium sp*, *Bacteroides sp* | Amoxicillin-clavulanic acid IV 150 mg/kg/day (maximum 4 g/day) in 3 or 4 divided doses | Cefotaxime IV 200 mg/kg/day (maximum 12 g/day) in 3 or 4 divided doses | Antibiotic therapy after puncture when possible.  Alternative antibiotics: Cefotaxime combined with Metronidazole or Clindamycin.  Oral switch after clinical (apyrexia, reduction in pain and size) and biological (C-reactive protein) improvement. |
|  |  |  | + Metronidazole IV 40 mg/kg/day (maximum 1.5 g/day) in 2 or 3 divided doses |  |
|  |  |  | or Clindamycin IV 40 mg/kg/day (maximum 2.4 g/day) in 4 divided doses |  |
| Purulent acute otitis media without conjonctivitis, **empiric treatment** | *Streptococcus pneumoniae*, *Haemophilus influenzae*  Other bacteria: *M. catarrhalis*, *S. pyogenes* (Group A streptococcus) | Amoxicillin PO 80-100 mg/kg/day (maximum 3 g/day) in 2 or 3 divided doses | Cefpodoxime PO 8 mg/kg/day (maximum 400 mg/day) in 2 divided doses | The use of Ceftriaxone has to be limited for exceptional situations of digestive intolerance or strong suspicion of resistant pneumococcus. |
|  |  |  | or Ceftriaxone IV or IM 50 mg/kg/day (maximum 1 g/day) in 1 daily dose |  |
| Purulent acute otitis media with conjunctivitis, **empiric treatment** | *Haemophilus influenzae* | Amoxicillin-clavulanic acid PO 80 mg/kg/day (maximum 3 g/day) in 2 or 3 divided doses | Cefpodoxime PO 8 mg/kg/day (maximum 400 mg/day) in 2 divided doses |  |
| Purulent acute otitis media after failure of first line treatment, **empiric treatment** | *Haemophilus influenzae*, *Streptococcus pneumoniae* | Failure after first treatment with Amoxicillin: Amoxicillin-clavulanic acid PO 80 mg/kg/day (maximum 3 g/day) in 2 or 3 divided doses | Failure after first treatment with Amoxicillin: Cefpodoxime PO 8 mg/kg/day (maximum 400 mg/day) in 2 divided doses | Definition of failure: persistence or reappearance of clinical signs during treatment or at least 72 hours after treatment stopped.  The use of Ceftriaxone has to be limited to exceptional situations of absolute gut intolerance or failure after treatment with Amoxicillin-clavulanic acid. |
|  |  | Failure after first treatment with Amoxicillin-clavulanic acid: Ceftriaxone IV or IM 50 mg/kg/day (maximum 1 g/day) in 1 daily dose, if possible after tympanocentesis |  |  |
|  |  | Failure after first treatment with Cefpodoxime: Amoxicillin PO 150 mg/kg/day (maximum 6 g/day) in 3 divided doses |  |  |
| Acute bacterial parotitis in newborns, without meningitis, **empiric treatment** | *Streptococcus agalactiae*, MSSA | Amoxicillin-clavulanic acid IV 100-150 mg/kg/day in 3 divided doses |  | Oral switch after clinical (apyrexia, reduction in size) and biological (C-reactive protein) improvement. |
|  |  | +/- Gentamicin IV 5 mg/kg/day in 1 daily dose for 48 hours, if septicaemic form |  |  |
| Acute bacterial parotitis (excluding newborns), **empiric treatment** | MSSA | Amoxicillin-clavulanic acid PO 80 mg/kg/day (maximum 4 g/day) in 3 divided doses | Clindamycin PO 30-40 mg/kg/day (maximum 2.4 g/day) in 3 divided doses |  |
|  |  | or Amoxicillin-clavulanic acid IV 100-150 mg/kg/day (maximum 4 g/day) in 3 or 4 divided doses |  |  |
| Respiratory |  |  |  |  |
| Community-acquired pneumonia, **empiric treatment** | *Streptococcus pneumoniae* | Amoxicillin PO or IV 80-100 mg/kg/day (maximum 3 g/day) in 3 divided doses | In the case of allergy to penicillin:  3rd-generation cephalosporin: Ceftriaxone IV or IM 50 mg/kg/day (maximum 2 g/day) in 1 daily dose; or Cefotaxime IV 100 mg/kg/day (maximum 6 g/day) in 3 divided doses | Administration of Amoxicillin in 2 doses/day can be considered after clinical improvement. |
|  |  |  | or after 6 years: Pristinamycin PO 50 mg/kg/day (maximum 4 g/day) in 2 divided doses |  |
| Community-acquired pneumonia in children <3 months old or with purulent acute otitis media and conjunctivitis, **empiric treatment** | *Streptococcus pneumoniae*, *Haemophilus influenzae* | Amoxicillin-clavulanic acid IV 100 mg/kg/day (maximum 6 g/day) in 3 divided doses |  |  |
|  |  | or Amoxicillin-clavulanic acid PO 80 mg/kg/day (maximum 3 g/day) in 3 divided doses |  |  |
| Community-acquired pneumonia in children with sickle cell disease, **empiric treatment** | *Salmonella sp*, *Streptococcus pneumoniae* | 3rd-generation cephalosporin: Cefotaxime IV 100 mg/kg/day (maximum 6 g/day) in 3 divided doses; or Ceftriaxone IV or IM 50 mg/kg/day (maximum 2 g/day) in 1 daily dose |  |  |
| Atypical community-acquired pneumonia | *Mycoplasma pneumoniae*, *Chlamydia pneumoniae* | Clarithromycin PO 15 mg/kg/day (maximum 1 g/day) in 2 divided doses | Josamycin PO 50 mg/kg/day (maximum 2 g/day) in 2 divided doses | The diagnosis should be raised when: child is older than 3 years; progressive installation; good general condition; absence of high C-reactive protein; failure of Amoxicillin; no pleural effusion.  Cross allergy between macrolides is rare. |
|  |  |  | or Azithromycin (if pneumococcus is excluded) PO 20 mg/kg/day (maximum 500 mg/day) in 1 daily dose |  |
|  |  |  | or after 6 years: Pristinamycin PO 50 mg/kg/day (maximum 4 g/day) in 2 divided doses |  |
| Aspiration pneumonia, **empiric treatment** | *Streptococcus pneumoniae*, Anaerobic bacteria (*Fusobacterium*, *Peptostreptococcus*, *Bacteroides*) | Amoxicillin-clavulanic acid IV 100 mg/kg/day (maximum 6 g/day) in 3 divided doses | 3rd-generation cephalosporin: Ceftriaxone IV or IM 50 mg/kg/day (maximum 2 g/day) in 1 daily dose; or Cefotaxime IV 100 mg/kg/day (maximum 6 g/day) in 3 divided doses |  |
|  |  | or Amoxicillin-clavulanic acid PO 80 mg/kg/day (maximum 3 g/day) in 3 divided doses | + Metronidazole IV or PO 30 mg/kg/day (maximum 1.5 g/day) in 3 divided doses |  |
| Non-severe community-acquired pleural empyema, **empiric treatment** | *Streptococcus pneumoniae*, *Streptococcus pyogenes* (Group A streptococcus), MSSA | Amoxicillin-clavulanic acid IV 150 mg/kg/day (maximum 6 g/day) in 3 divided doses | Cefotaxime IV 200 mg/kg/day (maximum 24 g/day) in 3 divided doses | Analysis of pleural effusion is necessary for bacterial documentation.  Persistence of fever beyond the 3rd day is not a sign of antibiotic treatment failure. Before any change in antibiotic treatment, consultation with an infectious disease specialist is recommended.  Switch to oral antibiotic according to clinical improvement. |
| Non-severe community-acquired pleural empyema due to MSSA | MSSA | Cloxacillin IV 200 mg/kg/day (maximum 12 g/day) in 4 divided doses | Cefamandole IV 150 mg/kg/day (maximum 3 g/day) in 3 divided doses | Switch to oral antibiotic according to clinical improvement.  Oral switch: Amoxicillin-clavulanic acid or Cefadroxil. |
| Non-severe community-acquired pleural empyema due to MRSA | MRSA | Vancomycin IV 60 mg/kg/day (maximum 4 g/day) in 4 divided doses or by continuous infusion (after loading dose of 15 mg/kg (maximum 2 g)) | Linezolid IV 30 mg/kg/day (maximum 1.2 g/day) in 2 divided doses | Preferred antibiotics: Vancomycin combined with Clindamycin or Rifampicin.  Switch to oral antibiotic according to clinical improvement. |
|  |  | + Clindamycin IV 40 mg/kg/day (maximum 2.4 g/day) in 3 divided doses |  |  |
|  |  | or Rifampicin IV 20 mg/kg/day (maximum 900 mg/day for <70 kg, 1200 mg/day for ≥70 kg) in 2 divided doses |  |  |
| Severe community-acquired pneumonia or pleural empyema (signs of severity: haemoptysis, leukopenia, toxinic signs [necrotising or rash], severe sepsis or septic shock), **empiric treatment** | MSSA, MRSA, *Streptococcus pyogenes* (Group A streptococcus) | Amoxicillin-clavulanic acid IV 150 mg/kg/day (maximum 6 g/day) in 4 divided doses | Cefotaxime IV 200 mg/kg/day (maximum 24 g/day) in 4 divided doses | Drainage is necessary if pleural effusion. |
|  |  | + Vancomycin IV 60 mg/kg/day (maximum 4 g/day) in 4 divided doses or by continuous infusion (after loading dose of 15 mg/kg (maximum 2 g)) | + Vancomycin IV 60 mg/kg/day (maximum 4 g/day) in 4 divided doses or by continuous infusion (after loading dose of 15 mg/kg (maximum 2 g)) |  |
|  |  | + Clindamycin IV 40 mg/kg/day (maximum 2.4 g/day) in 3 divided doses | + Clindamycin IV 40 mg/kg/day (maximum 2.4 g/day) in 3 divided doses |  |
| Severe community-acquired pneumonia or pleural empyema (signs of severity: haemoptysis, leukopenia, toxinic signs [necrotising or rash], severe sepsis or septic shock) due to Methicillin-sensitive and Erythromycin-sensitive PVL-positive *Staphylococcus aureus* | Methicillin-susceptible and Erythromycin-susceptible PVL-positive *Staphylococcus aureus* | Cloxacillin IV 200 mg/kg/day (maximum 12 g/day) in 4 divided doses | Vancomycin IV 60 mg/kg/day (maximum 4 g/day) in 4 divided doses or by continuous infusion (after loading dose of 15 mg/kg (maximum 2 g)) |  |
|  |  | + Clindamycin IV 40 mg/kg/day (maximum 2.4 g/day) in 3 divided doses | + Clindamycin IV 40 mg/kg/day (maximum 2.4 g/day) in 3 divided doses |  |
| Severe community-acquired pneumonia or pleural empyema (signs of severity: haemoptysis, leukopenia, toxinic signs [necrotising or rash], severe sepsis or septic shock) due to Methicillin-sensitive and Erythromycin-resistant PVL-positive *Staphylococcus aureus* | Methicillin-susceptible and Erythromycin-resistant PVL-positive *Staphylococcus aureus* | Cloxacillin IV 200 mg/kg/day (maximum 12 g/day) in 4 divided doses | Vancomycin IV 60 mg/kg/day (maximum 4 g/day) in 4 divided doses or by continuous infusion (after loading dose of 15 mg/kg (maximum 2 g)) | For Erythomycin-resistant strain, do not use Clindamycin due to the risk of inducible resistance.  Cloxacillin or Vancomycin combined with Rifampicin or Linezolid. |
|  |  | + Rifampicin IV 20 mg/kg/day (maximum 900 mg/day for <70 kg, 1200 mg/day for ≥70 kg) in 2 divided doses | + Rifampicin IV 20 mg/kg/day (maximum 900 mg/day for <70 kg, 1200 mg/day for ≥70 kg) in 2 divided doses |  |
|  |  | or Linezolid IV 30 mg/kg/day (maximum 1.2 g/day) in 2 divided doses | or Linezolid IV 30 mg/kg/day (maximum 1.2 g/day) in 2 divided doses |  |
| Severe community-acquired pneumonia or pleural empyema (signs of severity: haemoptysis, leukopenia, toxinic signs [necrotising or rash], severe sepsis or septic shock) due to Methicillin-resistant PVL-positive *Staphylococcus aureus* | Methicillin-resistant PVL-positive *Staphylococcus aureus* | Vancomycin IV 60 mg/kg/day (maximum 4 g/day) in 4 divided doses or by continuous infusion (after loading dose of 15 mg/kg (maximum 2 g)) | Linezolid IV 30 mg/kg/day (maximum 1.2 g/day) in 2 divided doses |  |
|  |  | + Clindamycin IV 40 mg/kg/day (maximum 2.4 g/day) in 3 divided doses |  |  |
| Early-onset VAP (<5 days of invasive ventilation) or non-ventilator HAP (<5 days of hospital stay), with no severe sepsis/septic shock and no risk factors for non-fermenting GNB^a^, **empiric treatment** | Community bacteria | Amoxicillin-clavulanic acid IV 100 mg/kg/day (maximum 6 g/day) in 3 divided doses | In the case of allergy to ß-lactam:  Levofloxacin IV 20 mg/kg/day if <5 years, 10 mg/kg/day if ≥5 years (maximum 1 g/day) in 2 divided doses | Oral switch after clinical and biological improvement if child is able to tolerate oral medication and oral antibiotic treats the identified or expected pathogen. |
|  |  | or 3rd-generation cephalosporin: Cefotaxime IV 100 mg/kg/day (maximum 6 g/day) in 3 divided doses; or Ceftriaxone IV 50 mg/kg/day (maximum 2 g/day) in 1 daily dose |  |  |
| Early-onset VAP (<5 days of invasive ventilation) or non-ventilator HAP (<5 days of hospital stay), with severe sepsis or septic shock and with no risk factors for non-fermenting GNB^a^, **empiric treatment** | Community bacteria | Amoxicillin-clavulanic acid IV 100 mg/kg/day (maximum 6 g/day) in 3 divided doses | In the case of allergy to ß-lactam:  Levofloxacin IV 20 mg/kg/day if <5 years, 10 mg/kg/day if ≥5 years (maximum 1 g/day) in 2 divided doses | Preferred antibiotics: aminoglycoside combined with Amoxicillin-clavulanic acid or 3rd-generation cephalosporin.  Oral switch after clinical and biological improvement if child is able to tolerate oral medication and oral antibiotic treats the identified or expected pathogen. |
|  |  | or 3rd-generation cephalosporin: Cefotaxime IV 100 mg/kg/day (maximum 6 g/day) in 3 divided doses; or Ceftriaxone IV 50 mg/kg/day (maximum 2 g/day) in 1 daily dose |  |  |
|  |  | + Aminoglycoside for at least 48 hours: Gentamicin IV 5 mg/kg/day in 1 daily dose; or Amikacin 15 mg/kg/day in 1 daily dose | + Aminoglycoside for at least 48 hours: Gentamicin IV 5 mg/kg/day in 1 daily dose; or Amikacin 15 mg/kg/day in 1 daily dose |  |
| Early-onset VAP (<5 days of invasive ventilation) or non-ventilator HAP (<5 days of hospital stay), with at least one of the risk factors for non-fermenting GNB^a^, **empiric treatment** | Community bacteria, non-fermenting GNB | Ceftazidime IV 200 mg/kg/day (maximum 6 g/day) in 4 divided doses | In the case of allergy to ß-lactam:  Aztreonam IV 80 mg/kg/day (maximum 6 g/day) in 3 divided doses | Preferred antibiotics: aminoglycoside combined with Ceftazidime or Cefepime or Piperacillin-tazobactam or Meropenem. Favour the use of Amikacin over Gentamicin due to enhanced efficacy against non-fermenting GNB.  For Piperacillin-tazobactam, the daily dose is 400 mg/kg/day to be active against *P. aeruginosa*.  Oral switch after clinical and biological improvement if child is able to tolerate oral medication and oral antibiotic treats the identified or expected pathogen. |
|  |  | or Cefepime IV 150 mg/kg/day (maximum 6 g/day) in 3 divided doses |  |  |
|  |  | or Piperacillin-tazobactam IV 400 mg/kg/day (maximum 4 g q6-8h) in 3 or 4 divided doses | + Clindamycin IV 40 mg/kg/day (maximum 600 mg q6-8h) in 3 or 4 divided doses |  |
|  |  | or Meropenem IV 60 mg/kg/day (maximum 6 g/day) in 3 divided doses, if recent colonisation or infection with ESBL Enterobacteriaceae |  |  |
|  |  | + Amikacin IV 15-30 mg/kg/day in 1 daily dose, until pathogen identification and susceptibility testing results are available |  |  |
| Late-onset VAP (≥5 days of invasive ventilation) or non-ventilator HAP (≥5 days of hospital stay), **empiric treatment** | Community bacteria, non-fermenting GNB | Ceftazidime IV 200 mg/kg/day (maximum 6 g/day) in 4 divided doses | In the case of allergy to ß-lactam:  Aztreonam IV 80 mg/kg/day (maximum 6 g/day) in 3 divided doses | Preferred antibiotics: aminoglycoside or fluoroquinolone combined with Ceftazidime or Cefepime or Piperacillin-tazobactam or Meropenem. Favour the use of aminoglycosides over fluoroquinolones to limit emergence of MDR bacteria. Favour the use of Amikacin over Gentamicin due to enhanced efficacy against non-fermenting GNB.  For Piperacillin-tazobactam, the daily dose is 400 mg/kg/day to be active against *P. aeruginosa*.  For Ciprofloxacin, the daily dose is 45 mg/kg/day to be active against *P. aeruginosa*.  Oral switch after clinical and biological improvement if child is able to tolerate oral medication and oral antibiotic treats the identified or expected pathogen. |
|  |  | or Cefepime IV 150 mg/kg/day (maximum 6 g/day) in 3 divided doses |  |  |
|  |  | or Piperacillin-tazobactam IV 400 mg/kg/day (maximum 4 g q6-8h) in 3 or 4 divided doses | + Clindamycin IV 40 mg/kg/day (maximum 600 mg q6-8h) in 3 or 4 divided doses |  |
|  |  | or Meropenem IV 60 mg/kg/day (maximum 6 g/day) in 3 divided doses, if recent colonisation or infection with ESBL Enterobacteriaceae |  |  |
|  |  | + Amikacin IV 15-30 mg/kg/day in 1 daily dose, until pathogen identification and susceptibility testing results are available |  |  |
|  |  | or Ciprofloxacin IV 45 mg/kg/day (maximum 400 mg q8h) in 3 divided doses |  |  |
| HAP, with at least one of the risk factors for MRSA^b^, **empiric treatment** | MRSA | + Vancomycin IV 40 mg/kg/day (maximum 4 g/day) in 4 divided doses or by continuous infusion (after loading dose of 15 mg/kg (maximum 2 g)) |  |  |
|  |  | or Linezolid IV 30 mg/kg/day (maximum 1.2 g/day) in 2 divided doses |  |  |
| Intra-abdominal |  |  |  |  |
| Necrotising enterocolitis, with no or only one risk factor for MDR infection, **empiric treatment** | Enterobacteriaceae, Anaerobic bacteria | Piperacillin-tazobactam IV 300 mg/kg/day (maximum 4 g q8h) in 3 divided doses |  | No oral switch. |
|  |  | + Aminoglycoside for at least 48 hours: Amikacin IV 30 mg/kg/day in 1 daily dose; or Gentamicin IV 5-8 mg/kg/day in 1 daily dose |  |  |
| Necrotising enterocolitis, with at least two of the risk factors for MDR infection, **empiric treatment** | ESBL Enterobacteriaceae | Meropenem IV 60 mg/kg/day (maximum 3 g/day) in 3 divided doses |  | No oral switch. |
|  |  | + Aminoglycoside for at least 48 hours: Amikacin IV 30 mg/kg/day in 1 daily dose; or Gentamicin IV 5-8 mg/kg/day in 1 daily dose |  |  |
| Bacterial colitis, **empiric treatment** | *Salmonella*, *Campylobacter*, *Yersinia*, *Shigella*, *E. coli* | Ceftriaxone IV 50 mg/kg/day (maximum 2 g/day) in 1 daily dose |  | Oral switch after clinical improvement. |
| Bacterial gastroenteritis due to *Campylobacter jejuni* | *Campylobacter jejuni* | Azithromycin PO 20 mg/kg/day (maximum 500 mg/day) in 1 daily dose | Ciprofloxacin PO 20-30 mg/kg/day (maximum 1500 mg/day) in 2 divided doses |  |
| Typhoid fever without complications | *Salmonella typhi* | Ceftriaxone IV 75 mg/kg/day (maximum 4 g/day) in 2 divided doses | Ciprofloxacin IV 20 mg/kg/day (maximum 400 mg X 2/day) in 2 divided doses | Oral switch after clinical improvement. |
| Community-acquired peritonitis, with no signs of severity, **empiric treatment** | Enterobacteriaceae, Anaerobic bacteria | Amoxicillin-clavulanic acid IV 150 mg/kg/day (maximum 6 g/day) in 3 divided doses | In the case of allergy to penicillin:  3rd-generation cephalosporin: Cefotaxime IV 100 mg/kg/day (maximum 6 g/day) in 3 divided doses; or Ceftriaxone IV 50 mg/kg/day (maximum 2 g/day) in 1 daily dose | No oral switch. |
|  |  | + Aminoglycoside for a maximum of 5 days (de-escalation once culture and susceptibility results are available): Amikacin IV 20 mg/kg/day in 1 daily dose; or Gentamicin IV 5-8 mg/kg/day in 1 daily dose |  |  |
|  |  |  | + Metronidazole IV 30 mg/kg/day (maximum 1.5 g/day) in 3 divided doses |  |
|  |  |  | In the case of allergy to ß-lactam:  Levofloxacin IV 8-10 mg/kg q12h if <5 years, 10 mg/kg q24h if ≥5 years (maximum 750 mg/day) |  |
|  |  |  | + Metronidazole IV 30 mg/kg/day (maximum 1.5 g/day) in 3 divided doses |  |
|  |  |  | + Aminoglycoside until pathogen identification and susceptibility testing results are available: Amikacin IV 20 mg/kg/day in 1 daily dose; or Gentamicin IV 5-8 mg/kg/day in 1 daily dose |  |
| Severe community-acquired peritonitis (signs of severity: presence of at least two of the following manifestations in the absence of another cause: tachycardia or hypotension related to the infection and requiring fluid loading >20 mL/kg; serum lactate > 2 mmol/L; urine output < 1 mL/kg/h despite appropriate IV fluid therapy; respiratory distress or hypoxia in the absence of pneumonia; acute kidney failure; hepatic failure or high serum bilirubin in the absence of liver disease; thrombocytopenia < 100 000/mm3 or prothrombin levels < 50%), **empiric treatment** | Enterobacteriaceae, non-fermenting GNB, Anaerobic bacteria, Enterococci | Piperacillin-tazobactam IV 300 mg/kg/day (maximum 4 g q8h) in 3 divided doses | In the case of allergy to ß-lactam:  Levofloxacin IV 8-10 mg/kg q12h if <5 years, 10 mg/kg q24h if ≥5 years (maximum 750 mg/day) | No oral switch. |
|  |  | + Aminoglycoside until pathogen identification and susceptibility testing results are available: Amikacin IV 30 mg/kg/day in 1 daily dose; or Gentamicin IV 5-8 mg/kg/day in 1 daily dose |  |  |
|  |  |  | + Metronidazole IV 30 mg/kg/day (maximum 1.5 g/day) in 3 divided doses |  |
|  |  |  | + Aminoglycoside until pathogen identification and susceptibility testing results are available: Amikacin IV 30 mg/kg/day in 1 daily dose; or Gentamicin IV 5-8 mg/kg/day in 1 daily dose |  |
| Healthcare-associated peritonitis, with no signs of severity, **empiric treatment** | Enterobacteriaceae, non-fermenting GNB, Anaerobic bacteria, Enterococci | Piperacillin-tazobactam IV 300 mg/kg/day (maximum 4 g q8h) in 3 divided doses | In the case of allergy to ß-lactam:  Ciprofloxacin IV 30 mg/kg/day (maximum 400 mg q8h) in 3 divided doses | No oral switch. |
|  |  | or Meropenem IV 60 mg/kg/day (maximum 3 g/day) in 3 divided doses, if presence of at least two of the risk factors for MDR peritonitis^c^ |  |  |
|  |  |  | + Metronidazole IV 30 mg/kg/day (maximum 1.5 g/day) in 3 divided doses |  |
|  |  |  | + Vancomycin IV 40 mg/kg/day (maximum 4 g/day) in 4 divided doses or by continuous infusion (after loading dose of 15 mg/kg (maximum 2 g)) |  |
|  |  |  | + Aminoglycoside until pathogen identification and susceptibility testing results are available: Amikacin IV 30 mg/kg/day in 1 daily dose; or Gentamicin IV 5-8 mg/kg/day in 1 daily dose |  |
| Severe healthcare-associated peritonitis (signs of severity: presence of at least two of the following manifestations in the absence of another cause: tachycardia or hypotension related to the infection and requiring fluid loading >20 mL/kg; serum lactate > 2 mmol/L; urine output < 1 mL/kg/h despite appropriate IV fluid therapy; respiratory distress or hypoxia in the absence of pneumonia; acute kidney failure; hepatic failure or high serum bilirubin in the absence of liver disease; thrombocytopenia < 100 000/mm3 or prothrombin levels < 50%), **empiric treatment** | Enterobacteriaceae, non-fermenting GNB, Anaerobic bacteria, Enterococci | Piperacillin-tazobactam IV 300 mg/kg/day (maximum 4 g q8h) in 3 divided doses | In the case of allergy to ß-lactam:  Ciprofloxacin IV 30 mg/kg/day (maximum 400 mg q8h) in 3 divided doses | Preferred antibiotics: Amikacin combined with Piperacillin-tazobactam or Meropenem.  No oral switch. |
|  |  | or Meropenem IV 60 mg/kg/day (maximum 3 g/day) in 3 divided doses, if presence of at least one of the risk factors for MDR peritonitis^c^ |  |  |
|  |  | + Amikacin IV 30 mg/kg/day in 1 daily dose, until pathogen identification and susceptibility testing results are available | + Metronidazole IV 30 mg/kg/day (maximum 1.5 g/day) in 3 divided doses |  |
|  |  |  | + Vancomycin IV 40 mg/kg/day (maximum 4 g/day) in 4 divided doses or by continuous infusion (after loading dose of 15 mg/kg (maximum 2 g)) |  |
|  |  |  | + Aminoglycoside until pathogen identification and susceptibility testing results are available: Amikacin IV 30 mg/kg/day in 1 daily dose; or Gentamicin IV 5-8 mg/kg/day in 1 daily dose |  |
| Healthcare-associated peritonitis, with risk factors for peritonitis due to Ampicillin-resistant Enterococci^d^, **empiric treatment** | Ampicillin-resistant Enterococci | + Vancomycin IV 40 mg/kg/day (maximum 4 g/day) in 4 divided doses or by continuous infusion (after loading dose of 15 mg/kg (maximum 2 g)) |  | No oral switch. |
| Urinary tract |  |  |  |  |
| Uncomplicated acute cystitis in pubescent girls, **empiric treatment** |  | Fosfomycin trometamol PO 3 g, single dose |  |  |
| Uncomplicated acute cystitis in prepubescent girls, **empiric treatment** | *Escherichia coli*  Other bacteria: *Proteus*, *Klebsiella sp*, Enterococci, *Staphylococcus saprophyticus* | Amoxicillin-clavulanic acid PO 80 mg/kg/day (maximum 3 g/day) in 3 divided doses | Trimethoprim-sulfamethoxazole PO 30 mg/kg/day (of sulfamethoxazole) (maximum 1.6 g/day) in 2 divided doses | If clinical resolution occurs with Amoxicillin-clavulanic acid, it is not necessary to adapt the treatment according to antibiotic susceptibility testing (high concentrations of clavulanic acid in urine inhibit the majority of β-lactamases).  If no favourable course with Amoxicillin-clavulanic acid, treatment must be switched according to pathogen identification and susceptibility testing results, in the following order: 1) Trimethoprim-sulfamethoxazole; 2) Cefixime. |
|  |  |  | or Cefixime PO 8 mg/kg/day (maximum 400 mg/day) in 2 divided doses |  |
| Catheter-associated acute cystitis, **empiric treatment** |  | 3rd-generation cephalosporin: Ceftriaxone IV 50 mg/kg/day (maximum 2 g/day) in 1 daily dose; or Cefotaxime IV 100 mg/kg/day (maximum 6 g/day) in 3 divided doses; de-escalation once culture and susceptibility results are available |  | In the case of risk factors for ESBL Enterobacteriaceae^e^, empiric therapy should be Piperacillin-tazobactam or a ß-lactam according to previous antibiotic susceptibility results, avoiding a carbapenem as much as possible. |
| Acute cystitis with risk of complications (any urinary tree abnormality, uropathy, recent urologic procedure, pregnancy, creatinine clearance <30 ml/min/1,73m2, or immunodepression), **empiric treatment** |  | 3rd-generation cephalosporin: Ceftriaxone IV 50 mg/kg/day (maximum 2 g/day) in 1 daily dose; or Cefotaxime IV 100 mg/kg/day (maximum 6 g/day) in 3 divided doses; de-escalation once culture and susceptibility results are available |  | In the case of risk factors for ESBL Enterobacteriaceae^e^, empiric therapy should be Piperacillin-tazobactam or a ß-lactam according to previous antibiotic susceptibility results, avoiding a carbapenem as much as possible. |
| Community-acquired acute pyelonephritis, with no signs of severity (severe sepsis or septic shock or organ dysfunction) and no urinary tract drainage (except bladder catheterisation), **empiric treatment** | *Escherichia col*i  Other bacteria: *Proteus*, *Klebsiella sp*, Enterococci, *Staphylococcus saprophyticus* | 3rd-generation cephalosporin: Cefotaxime IV 150 mg/kg/day (maximum 4.5 g/day) in 3 divided doses; or Ceftriaxone IV or IM 50 mg/kg/day (maximum 2 g/day) in 1 daily dose | In the case of allergy to ß-lactam: | Before 1 month old, Cefotaxime has to be used preferentially than Ceftriaxone.  Initial treatment is prescribed for 2–4 days, the duration required to obtain apyrexia and antibiotic susceptibility testing results.  Oral switch should be adapted to susceptibility testing results, in the following order: 1) Trimethoprim-sulfamethoxazole (for age ≥ 1 month old) 30 mg/kg/day of sulfamethoxazole in 2 divided doses; 2) Cefixime 8 mg/kg/day in 2 divided doses; 3) Ciprofloxacin 30 mg/kg/day in 2 divided doses (if sensitive to Nalidixic acid); 4) Amoxicillin for acute pyelonephritis due to *Enterococcus* or *Proteus sp* sensitive to Amoxicillin. |
|  |  |  | Amikacin IV 20-30 mg/kg/day (maximum 1 g/day) in 1 daily dose, until pathogen identification and susceptibility testing results are available |  |
| Community-acquired acute pyelonephritis, with organ dysfunction or urinary tract drainage (except bladder catheterisation) and with no severe sepsis/septic shock and no recent urinary tract colonisation or infection with ESBL Enterobacteriaceae during the previous 3 months, **empiric treatment** | *Escherichia coli*  Other bacteria: *Proteus*, *Klebsiella sp*, Enterococci, *Staphylococcus saprophyticus* | 3rd-generation cephalosporin: Cefotaxime IV 150 mg/kg/day (maximum 4.5 g/day) in 3 divided doses; or Ceftriaxone IV or IM 50 mg/kg/day (maximum 2 g/day) in 1 daily dose | In the case of allergy to ß-lactam:  Amikacin IV 20-30 mg/kg/day (maximum 1 g/day) in 1 daily dose, until pathogen identification and susceptibility testing results are available |  |
|  |  | + Amikacin IV 20-30 mg/kg/day (maximum 1 g/day) in 1 daily dose, until pathogen identification and susceptibility testing results are available |  |  |
| Community-acquired acute pyelonephritis, with organ dysfunction or urinary tract drainage (except bladder catheterisation), with no severe sepsis/septic shock and with recent urinary tract colonisation or infection with ESBL Enterobacteriaceae during the previous 3 months, **empiric treatment** | ESBL Enterobacteriaceae resistant to Piperacillin-tazobactam | Meropenem IV 60 mg/kg/day (maximum 6 g/day) in 3 divided doses |  |  |
|  |  | + Amikacin IV 20-30 mg/kg/day (maximum 1 g/day) in 1 daily dose, until pathogen identification and susceptibility testing results are available |  |  |
| Community-acquired acute pyelonephritis, with severe sepsis or septic shock and with no risk factors for ESBL Enterobacteriaceae^e^, **empiric treatment** | *Escherichia coli*  Other bacteria: *Proteus*, *Klebsiella sp*, Enterococci, *Staphylococcus saprophyticus* | 3rd-generation cephalosporin: Cefotaxime IV 150 mg/kg/day (maximum 4.5 g/day) in 3 divided doses; or Ceftriaxone IV or IM 50 mg/kg/day (maximum 2 g/day) in 1 daily dose | In the case of allergy to ß-lactam:  Amikacin IV 20-30 mg/kg/day (maximum 1 g/day) in 1 daily dose, until pathogen identification and susceptibility testing results are available |  |
|  |  | + Amikacin IV 20-30 mg/kg/day (maximum 1 g/day) in 1 daily dose, until pathogen identification and susceptibility testing results are available |  |  |
| Community-acquired acute pyelonephritis, with severe sepsis or septic shock and with at least one of the risk factors for ESBL Enterobacteriaceae^e^, **empiric treatment** | ESBL Enterobacteriaceae resistant to Piperacillin-tazobactam | Meropenem IV 60 mg/kg/day (maximum 6 g/day) in 3 divided doses |  |  |
|  |  | + Amikacin IV 20-30 mg/kg/day (maximum 1 g/day) in 1 daily dose, until pathogen identification and susceptibility testing results are available |  |  |
| Hospital-acquired acute pyelonephritis, with no signs of severity (severe sepsis or septic shock or organ dysfunction), no recent urinary tract colonisation or infection with *P. aeruginosa* during the previous 3 months and no risk factors for ESBL Enterobacteriaceae^e^, **empiric treatment** | *Escherichia coli*  Other bacteria: *Proteus*, *Klebsiella sp*, Enterococci, *Staphylococcus saprophyticus* | 3rd-generation cephalosporin: Cefotaxime IV 150 mg/kg/day (maximum 4.5 g/day) in 3 divided doses; or Ceftriaxone IV or IM 50 mg/kg/day (maximum 2 g/day) in 1 daily dose | In the case of allergy to ß-lactam or urinary tract drainage (except bladder catheterisation):  Amikacin IV 20-30 mg/kg/day (maximum 1 g/day) in 1 daily dose, until pathogen identification and susceptibility testing results are available |  |
|  |  |  |  |  |
| Hospital-acquired acute pyelonephritis, with no signs of severity (severe sepsis or septic shock or organ dysfunction) and with recent urinary tract colonisation or infection with ESBL Enterobacteriaceae or *P. aeruginosa* during the previous 3 months, **empiric treatment** | ESBL Enterobacteriaceae, *P. aeruginosa* | Choose a ß-lactam according to previous antibiotic susceptibility results, avoiding a carbapenem as much as possible | In the case of allergy to ß-lactam or urinary tract drainage (except bladder catheterisation):  Amikacin IV 20-30 mg/kg/day (maximum 1 g/day) in 1 daily dose, until pathogen identification and susceptibility testing results are available |  |
| Hospital-acquired acute pyelonephritis, with no signs of severity (severe sepsis or septic shock or organ dysfunction), with no recent urinary tract colonisation or infection with ESBL Enterobacteriaceae or *P. aeruginosa* during the previous 3 months and with at least one other of the risk factors for ESBL Enterobacteriaceae^e^, **empiric treatment** | ESBL Enterobacteriaceae sensitive to Piperacillin-tazobactam | Piperacillin-tazobactam IV 300 mg/kg/day (maximum 4 g q6-8h) in 3 or 4 divided doses | In the case of allergy to ß-lactam or urinary tract drainage (except bladder catheterisation):  Amikacin IV 20-30 mg/kg/day (maximum 1 g/day) in 1 daily dose, until pathogen identification and susceptibility testing results are available |  |
| Hospital-acquired acute pyelonephritis, with signs of severity (severe sepsis or septic shock or organ dysfunction), with no recent urinary tract colonisation or infection with *P. aeruginosa* during the previous 3 months and no risk factors for ESBL Enterobacteriaceae^e^, **empiric treatment** | *Escherichia coli*  Other bacteria: *Proteus*, *Klebsiella sp*, Enterococci, *Staphylococcus saprophyticus* | 3rd-generation cephalosporin: Cefotaxime IV 150 mg/kg/day (maximum 4.5 g/day) in 3 divided doses; or Ceftriaxone IV or IM 50 mg/kg/day (maximum 2 g/day) in 1 daily dose |  | Amikacin combined with Cefotaxime or Ceftriaxone or Piperacillin-tazobactam. |
|  |  | or Piperacillin-tazobactam IV 300 mg/kg/day (maximum 4 g q6-8h) in 3 or 4 divided doses |  |  |
|  |  | + Amikacin IV 20-30 mg/kg/day (maximum 1 g/day) in 1 daily dose, until pathogen identification and susceptibility testing results are available |  |  |
| Hospital-acquired acute pyelonephritis, with signs of severity (severe sepsis or septic shock or organ dysfunction) and with recent urinary tract colonisation or infection with *P. aeruginosa* during the previous 3 months, **empiric treatment** | *P. aeruginosa* | Choose a ß-lactam according to previous antibiotic susceptibility results, avoiding a carbapenem as much as possible |  |  |
|  |  | + Amikacin IV 20-30 mg/kg/day (maximum 1 g/day) in 1 daily dose, until pathogen identification and susceptibility testing results are available |  |  |
| Hospital-acquired acute pyelonephritis, with signs of severity (severe sepsis or septic shock or organ dysfunction) and with at least one of the risk factors for ESBL Enterobacteriaceae^e^, **empiric treatment** | ESBL Enterobacteriaceae resistant to Piperacillin-tazobactam | Meropenem IV 60 mg/kg/day (maximum 6 g/day) in 3 divided doses |  |  |
|  |  | + Amikacin IV 20-30 mg/kg/day (maximum 1 g/day) in 1 daily dose, until pathogen identification and susceptibility testing results are available |  |  |
| Acute pyelonephritis with Gram-positive cocci on direct examination | Enterococci | Amoxicillin IV 100 mg/kg/day (maximum 12 g/day) in 3 divided doses | Vancomycin IV 40 mg/kg/day (maximum 4 g/day) in 4 divided doses or by continuous infusion (after loading dose of 15 mg/kg (maximum 2 g)) |  |
| Central nervous system |  |  |  |  |
| Community-acquired bacterial meningitis in neonates aged 3-7 days old, **empiric treatment** | *Streptococcus agalactiae* (Group B streptococcus), *Escherichia coli* | Cefotaxime IV 200 mg/kg/day in 2 divided doses |  | No oral switch. |
|  |  | + Gentamicin IV 5 mg/kg/day in 1 daily dose for 48 hours |  |  |
|  | + *Listeria monocytogenes*, Enterococci | +/- Amoxicillin IV 200 mg/kg/day in 2 divided doses, if suspected Listeria meningitis and continue Gentamicin for 5 days if Listeria meningitis is confirmed |  |  |
| Community-acquired bacterial meningitis in neonates aged 8-28 days old, **empiric treatment** | *Streptococcus agalactiae* (Group B streptococcus), *Escherichia coli* | Cefotaxime IV 200 mg/kg/day in 3 or 4 divided doses |  | No oral switch. |
|  |  | + Gentamicin IV 5 mg/kg/day in 1 daily dose for 48 hours |  |  |
|  | + *Listeria monocytogenes*, Enterococci | +/- Amoxicillin IV 200 mg/kg/day in 3 divided doses, if suspected Listeria meningitis and continue Gentamicin for 5 days if Listeria meningitis is confirmed |  |  |
| Community-acquired bacterial meningitis in child 1-3 months old, with no risk factors for Listeria infection, **empiric treatment** | *Streptococcus agalactiae* (Group B streptococcus), *Escherichia coli*, *Streptococcus pneumoniae*, *Neisseria meningitidis*, *Haemophilus influenzae* | 3rd-generation cephalosporin: Cefotaxime IV 300 mg/kg/day (maximum 24 g/day) in 4 to 6 divided doses or by continuous infusion; or Ceftriaxone IV 100 mg/kg/day (maximum 4 g/day) in 1 or 2 divided doses | In the case of allergy to ß-lactam:  Vancomycin IV 60 mg/kg/day (maximum 4 g/day) in 4 divided doses or by continuous infusion (after loading dose of 15 mg/kg (maximum 2 g)) | Cefotaxime: the daily dose for the continuous infusion is initiated immediately after a loading dose of 50 mg/kg over one hour.  No oral switch. |
|  |  | + Gentamicin IV 5-8 mg/kg/day in 1 daily dose for 48 hours |  |  |
|  |  |  | + Rifampicin IV 40 mg/kg/day (maximum 300 mg q12h) in 2 divided doses |  |
|  |  |  | + Gentamicin IV 5-8 mg/kg/day in 1 daily dose for 48 hours |  |
| Community-acquired bacterial meningitis, in child ≥3 months old, with no risk factors for Listeria infection, **empiric treatment** | *Streptococcus pneumoniae*, *Neisseria meningitidis*, *Haemophilus influenzae* | 3rd-generation cephalosporin: Cefotaxime IV 300 mg/kg/day (maximum 24 g/day) in 4 to 6 divided doses or by continuous infusion; or Ceftriaxone IV 100 mg/kg/day (maximum 4 g/day) in 1 or 2 divided doses | In the case of allergy to ß-lactam:  Vancomycin IV 60 mg/kg/day (maximum 4 g/day) in 4 divided doses or by continuous infusion (after loading dose of 15 mg/kg (maximum 2 g)) | Cefotaxime: the daily dose for the continuous infusion is initiated immediately after a loading dose of 50 mg/kg over one hour.  No oral switch. |
|  |  |  | + Rifampicin IV 40 mg/kg/day (maximum 300 mg q12h) in 2 divided doses |  |
| Community-acquired bacterial meningitis (excluding neonates), with risk factors for Listeria infection, **empiric treatment** | *Streptococcus pneumoniae*, *Neisseria meningitidis*, *Haemophilus influenzae*, *Listeria monocytogenes* | 3rd-generation cephalosporin: Cefotaxime IV 300 mg/kg/day (maximum 24 g/day) in 4 to 6 divided doses or by continuous infusion; or Ceftriaxone IV 100 mg/kg/day (maximum 4 g/day) in 1 or 2 divided doses | In the case of allergy to ß-lactam:  Vancomycin IV 60 mg/kg/day (maximum 4 g/day) in 4 divided doses or by continuous infusion (after loading dose of 15 mg/kg (maximum 2 g)) | Cefotaxime: the daily dose for the continuous infusion is initiated immediately after a loading dose of 50 mg/kg over one hour.  No oral switch. |
|  |  | + Amoxicillin IV 200 mg/kg/day (maximum 12 g/day) in 4 to 6 divided doses or by continuous infusion |  |  |
|  |  | + Gentamicin IV 5-8 mg/kg/day in 1 daily dose for 48 hours (continue Gentamicin for 5 days if Listeria meningitis is confirmed) | + Rifampicin IV 40 mg/kg/day (maximum 300 mg q12h) in 2 divided doses |  |
|  |  |  | + Trimethoprim-sulfamethoxazole IV 40 mg/kg/day (of sulfamethoxazole) (maximum 2.4 g/day) in 2 divided doses |  |
| *Klebsiella sp* meningitis | *Klebsiella sp* | Choose the antibiotic according to susceptibility testing results |  | No oral switch. |
| *Listeria monocytogenes* meningitis | *Listeria monocytogenes* | Amoxicillin IV 200 mg/kg/day (maximum 12 g/day) in 4 to 6 divided doses or by continuous infusion |  | No oral switch. |
|  |  | + Gentamicin IV 5-8 mg/kg/day in 1 daily dose for 5 days |  |  |
| Healthcare-associated meningitis, **empiric treatment** | Methicillin-resistant Staphylococcus, non-fermenting GNB | Vancomycin IV 60 mg/kg/day (maximum 4 g/day) in 4 divided doses or by continuous infusion (after loading dose of 15 mg/kg (maximum 2 g)) | In case of allergy to ß-lactam:  Vancomycin IV 60 mg/kg/day (maximum 4 g/day) in 4 divided doses or by continuous infusion (after loading dose of 15 mg/kg (maximum 2 g)) | Preferred antibiotics: Vancomycin combined with Ceftazidime or Cefepime or Meropenem.  No oral switch. |
|  |  | + Ceftazidime IV 200 mg/kg/day (maximum 6 g/day) in 4 divided doses |  |  |
|  |  | or Cefepime IV 150 mg/kg/day (maximum 6 g/day) in 3 divided doses | + Aztreonam IV 120 mg/kg/day (maximum 6 g/day) in 4 divided doses |  |
|  |  | or Meropenem IV 120 mg/kg/day (maximum 6 g/day) in 3 divided doses, if recent colonisation or infection with ESBL Enterobacteriaceae |  |  |
| Skin and soft tissue |  |  |  |  |
| Trauma wound heavily soiled or on local ischaemic terrain, without or with localised lesions (erythema and induration extending <5 cm from the wound edge, even if purulent lesion) and with no general signs, **empiric treatment** | *Staphylococcus*, *Streptococcus*, Anaerobic bacteria (including *Clostridium perfringens*), Enterobacteriaceae, *Bacillus cereus*, *Aeromonas* (wound in water) | Amoxicillin-clavulanic acid PO 80 mg/kg/day (maximum 3 g/day) in 3 divided doses | Pristinamycin PO 50 mg/kg/day (maximum 4 g/day) in 2 or 3 divided doses | Washing with soap and antiseptic. |
|  |  |  | or Clindamycin PO 30-40 mg/kg/day (maximum 1.8 g/day) in 3 divided doses |  |
|  |  |  | or Trimethoprim-sulfamethoxazole PO 30 mg/kg/day (of sulfamethoxazole) (maximum 1.6 g/day) in 2 divided doses |  |
| Trauma wound with extended lesions (erythema and induration extending >5 cm from the wound edge, lymphangitis) and with no general signs, **empiric treatment** | *Staphylococcus*, *Streptococcus*, Anaerobic bacteria (including *Clostridium perfringens*), Enterobacteriaceae, *Bacillus cereus*, *Aeromonas* (wound in water) | Amoxicillin-clavulanic acid PO 80 mg/kg/day (maximum 3 g/day) in 3 divided doses | Pristinamycin PO 50 mg/kg/day (maximum 4 g/day) in 2 or 3 divided doses | Washing with soap and antiseptic. |
|  |  |  | or Clindamycin PO 30-40 mg/kg/day (maximum 1.8 g/day) in 3 divided doses |  |
|  |  |  | or Trimethoprim-sulfamethoxazole PO 30 mg/kg/day (of sulfamethoxazole) (maximum 1.6 g/day) in 2 divided doses |  |
| Superficial surgical site infection, with extended lesions, **empiric treatment** | *Staphylococcus*, *Streptococcus*, Anaerobic bacteria (including *Clostridium perfringens*), Enterobacteriaceae, *Bacillus cereus*, *Aeromonas* (wound in water) | Amoxicillin-clavulanic acid PO 80 mg/kg/day (maximum 3 g/day) in 3 divided doses | Pristinamycin PO 50 mg/kg/day (maximum 4 g/day) in 2 or 3 divided doses | Local wound management. |
|  |  |  | or Clindamycin PO 30-40 mg/kg/day (maximum 1.8 g/day) in 3 divided doses |  |
|  |  |  | or Trimethoprim-sulfamethoxazole PO 30 mg/kg/day (of sulfamethoxazole) (maximum 1.6 g/day) in 2 divided doses |  |
| Trauma wound with general signs, **empiric treatment** |  | See non-necrotising bacterial dermohypodermitis |  | Washing with soap and antiseptic. |
| Non-necrotising bacterial dermohypodermitis (cellulitis, erysipelas, caused by bites), with no risk factors, no toxinic signs and no signs of severity, **empiric treatment** | *Streptococcus pyogenes* (Group A streptococcus), *Staphylococcus aureus*  Frequent co-infections | Amoxicillin-clavulanic acid PO 80 mg/kg/day (maximum 3 g/day) in 3 divided doses | Cefadroxil PO 100 mg/kg/day (maximum 3 g/day) in 3 divided doses | For non-necrotising bacterial dermohypodermitis, microbiological samples are only recommended in the following cases: human or animal bite; aquatic environment; travel in tropical zones; post-traumatic, healthcare-associated (peripheral venous catheters) or due to septic injection (intravenous drug addiction); purulent lesions; failure after first presumed appropriate antibiotic treatment; immunodepression; local or general signs of severity (suspicion of necrotising bacterial dermohypodermitis). |
|  |  |  | or Trimethoprim-sulfamethoxazole PO 30 mg/kg/day (of sulfamethoxazole) (maximum 1.6 g/day) in 2 divided doses |  |
|  |  |  | or Clindamycin PO 40 mg/kg/day (maximum 1.8 g/day) in 3 divided doses |  |
| Non-necrotising bacterial dermohypodermitis (cellulitis, erysipelas, caused by bites), with risk factors (alteration of general condition; age < 1 year old; immunodepression; extended or rapidly evolving lesion; failure of oral antibiotics; presumption of secondary location [e.g., arthritis]; poor treatment compliance), with no toxinic signs and no signs of severity (see comments), **empiric treatment** | *Streptococcus pyogenes* (Group A streptococcus), *Staphylococcus aureus*  Frequent co-infections | Amoxicillin-clavulanic acid IV 100 mg/kg/day (maximum 6 g/day) in 3 divided doses | Cefamandole IV 150 mg/kg/day (maximum 6 g/day) in 3 or 4 divided doses | Signs of severity = severe sepsis /septic shock or toxic shock; severe pain that seems disproportional to local signs, impotence; local signs of severity (ecchymoses, crepitus indicating gas in the tissues, local hypoesthesia or anaesthesia, induration extending beyond the cutaneous erythema, skin necrosis); rapid extension of local signs in a few hours; worsening of local signs within 24 to 48 hours after initiating first antibiotic therapy, despite appropriate treatment.  Oral switch after clinical improvement (apyrexia, reduction in erythema), usually after 2-3 days.  In the case of signs of severity, treat as necrotising bacterial dermohypodermitis. |
|  |  |  | or Cefuroxime IV 100 mg/kg/day (maximum 3 g/day) in 3 or 4 divided doses |  |
|  |  |  | or in the case of allergy to cephalosporins:  Clindamycin IV 40 mg/kg/day (maximum 2.4 g/day) in 3 or 4 divided doses |  |
| Non-necrotising bacterial dermohypodermitis (cellulitis, erysipelas, caused by bites), with toxinic signs or severe sepsis or septic shock, **empiric treatment** | *Streptococcus pyogenes* (Group A streptococcus), *Staphylococcus aureus*  Frequent co-infections | Amoxicillin-clavulanic acid IV 150 mg/kg/day (maximum 6 g/day) in 3 or 4 divided doses | Cefuroxime IV 100 mg/kg/day (maximum 3 g/day) in 3 or 4 divided doses | Oral switch after clinical improvement (apyrexia, reduction in erythema).  In the case of allergy to cephalosporins or MRSA: advice from infectious disease specialist. |
|  |  | + Clindamycin IV 40 mg/kg/day (maximum 2.4 g/day) in 3 or 4 divided doses | + Clindamycin IV 40 mg/kg/day (maximum 2.4 g/day) in 3 or 4 divided doses |  |
|  |  | +/- Gentamicin IV 5-8 mg/kg/day in 1 daily dose for at least 48 hours, if severe sepsis or septic shock | +/- Gentamicin IV 5-8 mg/kg/day in 1 daily dose for at least 48 hours, if severe sepsis or septic shock |  |
| Acute staphylococcus epidermolysis with local detachments (< 20%) and moderate or absent general signs | *Staphylococcus aureus* (exfoliatin-producing) | Amoxicillin-clavulanic acid PO 80 mg/kg/day (maximum 3 g/day) in 3 divided doses | Cefadroxil PO 100 mg/kg/day (maximum 3 g/day) in 3 divided doses |  |
|  |  |  | or Trimethoprim-sulfamethoxazole PO 30 mg/kg/day (of sulfamethoxazole) (maximum 1.6 g/day) in 2 divided doses |  |
| Acute staphylococcus epidermolysis with extended lesions or general signs (staphylococcal scaled skin syndrome) | *Staphylococcus aureus* (exfoliatin-producing) | Cloxacillin IV 200 mg/kg/day (maximum 12 g/day) in 4 divided doses | Cefuroxime IV 100 mg/kg/day (maximum 3 g/day) in 3 or 4 divided doses |  |
|  |  |  | or Clindamycin IV 40 mg/kg/day (maximum 2.4 g/day) in 3 or 4 divided doses |  |
|  |  | Oral switch:  Amoxicillin-clavulanic acid PO 80 mg/kg/day (maximum 3 g/day) in 3 divided doses |  |  |
|  |  |  | Oral switch:  Cefadroxil PO 100 mg/kg/day (maximum 3 g/day) in 3 divided doses |  |
|  |  |  | or Trimethoprim-sulfamethoxazole PO 30 mg/kg/day (of sulfamethoxazole) (maximum 1.6 g/day) in 2 divided doses |  |
| Pilonidal cyst infection | *Staphylococcus*, Enterobacteriaceae, Anaerobic bacteria | Amoxicillin-clavulanic acid IV 100 mg/kg/day (maximum 6 g/day) in 3 divided doses |  |  |
| Bones and joints |  |  |  |  |
| Healthcare-associated osteitis, **empiric treatment** | Methicillin-resistant Staphylococcus, non-fermenting GNB | Vancomycin IV 60 mg/kg/day (maximum 4 g/day) in 4 divided doses or by continuous infusion (after loading dose of 15 mg/kg (maximum 2 g)) | Advice from infectious disease specialist | Vancomycin combined with Ceftazidime or Cefepime Piperacillin-tazobactam or Meropenem. |
|  |  | + Ceftazidime IV 200 mg/kg/day (maximum 6 g/day) in 4 divided doses |  |  |
|  |  | or Cefepime IV 150 mg/kg/day (maximum 6 g/day) in 3 divided doses |  |  |
|  |  | or Piperacillin-tazobactam IV 300 mg/kg/day (maximum 4 g q6-8h) in 3 or 4 divided doses |  |  |
|  |  | or Meropenem(2) IV 60 mg/kg/day (maximum 6 g/day) in 3 divided doses, if recent colonisation or infection with ESBL Enterobacteriaceae |  |  |
| Cardiovascular |  |  |  |  |
| Postsurgical mediastinitis, **empiric treatment** | Methicillin-resistant Staphylococcus, non-fermenting GNB | Piperacillin-tazobactam IV 300 mg/kg/day (maximum 4 g q8h) in 3 divided doses |  | Vancomycin combined with Piperacillin-tazobactam or Meropenem.  No oral switch. |
|  |  | or Meropenem IV 60 mg/kg/day (maximum 6 g/day) in 3 divided doses, if recent colonisation or infection with ESBL Enterobacteriaceae |  |  |
|  |  | + Vancomycin IV 40 mg/kg/day (maximum 4 g/day) in 4 divided doses or by continuous infusion (after loading dose of 15 mg/kg (maximum 2 g)) |  |  |
| Mediastinitis due to Methicillin-resistant coagulase-negative staphylococci | Methicillin-resistant coagulase-negative staphylococci | Vancomycin IV 40 mg/kg/day (maximum 4 g/day) in 4 divided doses or by continuous infusion (after loading dose of 15 mg/kg (maximum 2 g)) |  | No oral switch. |
| Purulent pericarditis, **empiric treatment** | *Staphylococcus*, *Streptococcus*, *Pneumococcus*, *Haemophilus sp*, *Neisseria meningitidis* | Amoxicillin-clavulanic acid IV 100 mg/kg/day (maximum 6 g/day) in 3 divided doses |  | Drainage is necessary.  Oral switch after clinical and biological improvement. |
| Myopericarditis due to *Leptospira* | *Leptospira sp* | Amoxicillin IV 100 mg/kg/day (maximum 6 g/day) in 3 divided doses |  | Oral switch after clinical and biological improvement. |

ESBL: Extended-Spectrum β-Lactamase producing; GNB: Gram-Negative Bacilli; HAP: Hospital-Acquired Pneumonia; IM: Intramuscular; IV: Intravenous; MDR: Multidrug-Resistant; MIC: Minimum Inhibitory Concentration; MRSA: Methicillin-Resistant *Staphylococcus aureus*; MSSA: Methicillin-Susceptible *Staphylococcus aureus*; PO: Per os; PVL: Panton and Valentine Leucocidin; VAP: Ventilator-Associated Pneumonia.

^a^Risk factors for non-fermenting gram-negative bacilli for hospital-acquired pneumonia: antibiotic therapy within 90 days prior to pneumonia, a hospital stay of 5 or more days preceding pneumonia, renal replacement therapy requirement prior to pneumonia, severe sepsis or septic shock, acute respiratory distress syndrome.

^b^Risk factors for MRSA: high local prevalence of MRSA, recent colonisation by MRSA, chronic skin lesions, chronic renal replacement therapy.

^c^Risk factors for MDR peritonitis: recent colonisation or infection with ESBL Enterobacteriaceae or Ceftazidime-resistant *P. aeruginosa* during the previous 3 months, regardless of the site; antibiotic therapy with a 3rd generation cephalosporin or fluoroquinolone (including a single dose) during the previous 3 months; hospitalisation in a country outside France during the previous 12 months; patient living in a nursing home or long-stay care AND with an indwelling catheter and/or gastrostomy; failure of broad-spectrum antibiotic therapy with a 3rd generation cephalosporin or fluoroquinolone or Piperacillin-tazobactam; early recurrence (< 2 weeks) of an infection treated by Piperacillin-tazobactam for at least 3 days.

^d^Risk factors for peritonitis due to Ampicillin-resistant Enterococci: hepatobiliary disease; liver transplant; antibiotic therapy with a cephalosporin or broad-spectrum ß-lactam during the previous 3 months.

^e^Risk factor for ESBL Enterobacteriaceae for urinary tract infections: recent urinary tract colonisation or infection with ESBL Enterobacteriaceae during the previous 3 months; antibiotic therapy with Amoxicillin-clavulanic acid, a 2nd or 3rd generation cephalosporin or fluoroquinolone (including a single dose) during the previous 3 months; travel in endemic regions for ESBL Enterobacteriaceae during the previous 3 months; patient living in a nursing home or long-stay care AND with an indwelling catheter and/or gastrostomy.

# Appendix 4.

**Local ICU Microbiological Data**

The enterobacterales resistance rate for at least one third-generation cephalosporin ranged from 7% to 45% depending on the centre and year of isolation, with 4% to 18% extended-spectrum β-lactamase (ESBL) producing isolates in four centres and 35% ESBL-producing isolates at only one centre for one year (Ile de la Réunion). Among Enterobacterales isolates, *E. coli* was the most isolated species and presented a third-generation cephalosporin resistance rate between 0% to 31%, with 0% to 25% ESBL-producing isolates in four centres and 27% ESBL-producing isolates at one centre for one year (Amiens). The enterobacterales resistance rate for carbapenems (excluding Imipenem for *Proteae*) was 0% to 14%.

*Pseudomonas aeruginosa* resistance rates spanned from 0% to 86% for Piperacillin-tazobactam, 0% to 40% for Ceftazidime and 0% to 16% for Meropenem or Imipenem depending on the centre and year; only one centre (Ile de la Réunion) presented a resistance rate above 26% for Piperacillin-tazobactam and 16% for Ceftazidime for one year. Among the few *Acinetobacter* strains isolated, only one was resistant to carbapenems.

Finally, among gram-positive pathogens, the methicillin-resistant *Staphylococcus aureus* rate ranged from 0% to 17% and although few *E. faecium* were isolated, no Vancomycin resistance was reported.
